# Dysregulated SREBP1c/miR-153 signaling induced by hypertriglyceridemia worsens acute pancreatitis and delays tissue repair

**DOI:** 10.1172/jci.insight.138584

**Published:** 2021-01-25

**Authors:** Juanjuan Dai, Mingjie Jiang, Yangyang Hu, Jingbo Xiao, Bin Hu, Jiyao Xu, Xiao Han, Shuangjun Shen, Bin Li, Zengkai Wu, Yan He, Yingchun Ren, Li Wen, Xingpeng Wang, Guoyong Hu

**Affiliations:** 1 Department of Gastroenterology and; 2 Shanghai Key Laboratory of Pancreatic Diseases, Shanghai General Hospital, Shanghai Jiaotong University School of Medicine, Shanghai, China.; 3 Department of Gastroenterology, The First Affiliated Hospital of Nanchang University, Nanchang, Jiangxi, China.; 4 Department of Emergency, Shanghai General Hospital, Shanghai Jiaotong University School of Medicine, Shanghai, China.

**Keywords:** Gastroenterology, Therapeutics, Epigenetics, Insulin, Molecular pathology

## Abstract

Severe acute pancreatitis (AP) is a life-threatening disease with up to 30% mortality. Therefore, prevention of AP aggravation and promotion of pancreatic regeneration are critical during the course and treatment of AP. Hypertriglyceridemia (HTG) is an established aggravating factor for AP that hinders pancreatic regeneration; however, its exact mechanism remains unclear. Using miRNA sequencing and further verification, we found that miRNA-153 (miR-153) was upregulated in the pancreas of HTG animal models and in the plasma of patients with HTG-AP. Increased miR-153 aggravated HTG-AP and delayed pancreatic repair via targeting TRAF3. Furthermore, miR-153 was transcriptionally suppressed by sterol regulatory element-binding transcription factor 1c (SREBP1c), which was suppressed by lipoprotein lipase malfunction-induced HTG. Overexpressing SREBP1c suppressed miR-153 expression, alleviated the severity of AP, and facilitated tissue regeneration in vivo. Finally, therapeutic administration of insulin also protected against HTG-AP via upregulating SREBP1c. Collectively, our results not only provide evidence that HTG leads to the development of more severe AP and hinders pancreatic regeneration via inducing persistent dysregulation of SREBP1c/miR-153 signaling, but also demonstrate that SREBP1c activators, including insulin, might be used to treat HTG-AP in patients.

## Introduction

Acute pancreatitis (AP) is a common and potentially life-threatening inflammatory disorder of the exocrine pancreas. Severe form of AP has a mortality up to 30% (1). As an established aggravating factor for AP, hypertriglyceridemia (HTG) is also the third most common cause of AP in the United States and the second most common cause of AP in China (2). Patients with severe and very severe HTG are prone to develop more severe AP (3, 4). Severe and very severe HTG also delays pancreatic recovery after AP (5) and increases the risk of developing recurrent and chronic pancreatitis (6, 7), which altogether can lead to other sequelae, including pancreatic exocrine insufficiency (8). However, effective drugs for treating HTG-AP are not yet readily available.

Severe and very severe HTG are primarily caused by mutations in genes associated with lipoprotein lipase (LPL), including *APOC2*, *APOA5*, *GPIHBP1,* and *LPL* itself in patients with primary HTG such as familial HTG (9). LPL is the rate-limiting enzyme for releasing triglyceride (TG) from circulating lipoproteins. The malfunction of LPL causes accumulation of massive TG in the plasma, which along with free fatty acids (FFAs) are considered as the main mechanism for increased AP severity (10–14). FFAs released from the hydrolysis of TGs by pancreatic lipase in the peripancreatic fat were found to cause local and systematic injury during AP (15, 16). Several studies also showed that FFAs could directly cause abnormal Ca^2+^ signals, mitochondrial dysfunction, and excessive ER stress in pancreatic acinar cells (10–12, 17). Accordingly, lipid-lowering therapies, including plasmapheresis, are used on patients with AP with severe and very severe HTG. Nonetheless, although plasmapheresis can rapidly reduce serum TG level, the therapeutic effects of plasmapheresis remain controversial (4, 18–23). However, insulin, another TG-lowering therapeutic molecule that has pleiotropic effects on lipid and glucose metabolism, has been reported to effectively alleviate HTG-AP (24, 25). These clinical observations indicate that other aggravating factors may exist after the clearance of TG/FFAs and demonstrate the urgent need to explore the existence of persistent pancreatic signaling changes caused by HTG that are responsible for increased AP severity.

miRNAs are a class of small noncoding RNAs that play vital roles in the regulation of virous physiological and pathological processes. Using miRNA sequencing and further verification, we found that miRNA-153 (miR-153) was specifically upregulated in the plasma of patients with HTG-AP and in 2 experimental HTG-AP models. Increased miR-153 worsened AP and delayed pancreatic repair via targeting TNF receptor–associated factor 3 (*Traf3*) in mice with LPL malfunction-induced HTG. Further investigation showed that miR-153 was transcriptionally suppressed by sterol regulatory element-binding transcription factor 1c (SREBP1c), which was inhibited by LPL malfunction-induced HTG. Both genetically overexpressing SREBP1c and pharmacologically activating SREBP1c by insulin rescued mice from HTG-AP and facilitated pancreatic repair via inhibiting miR-153 expression. Our data suggest that insulin infusion or other specific SREBP1c activators could also be effective therapeutic approaches for patients with AP with LPL malfunction-induced HTG.

## Results

### Upregulated miR-153 specifically induced by HTG increases pancreatitis severity in 2 models of AP.

**Figure 1:**
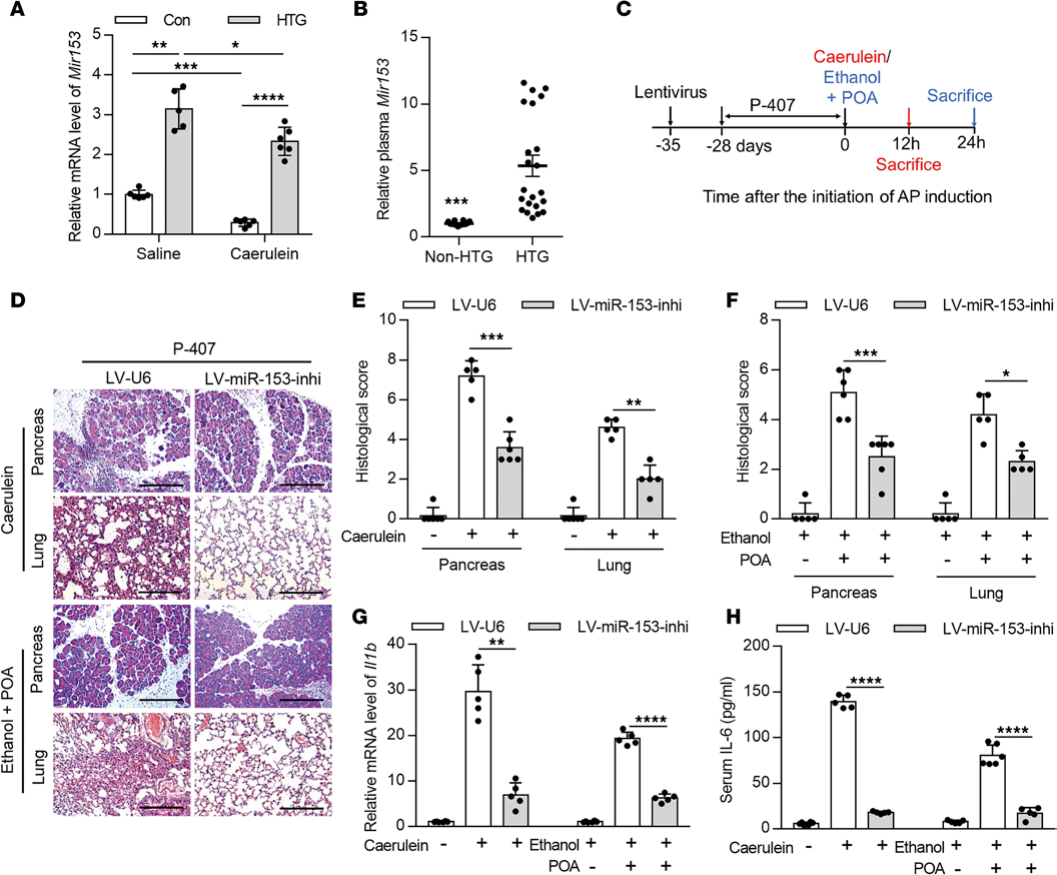
miR-153 is upregulated by HTG and aggravates AP. (**A**) qPCR quantification of *Mir153* level. *Rnu6* was used as an endogenous control. (**B**) qPCR quantification of *Mir153* level in the plasma of patients with AP. Ce_miR-39_1 was used as a spike in control. (**C**) Schematic of the experimental procedure to induce HTG-AP model in lentivirus-injected mice. (**D**) Representative images of H&E-stained pancreas and lung sections from HTG mice with cerulein-induced (top) or alcoholic (bottom) pancreatitis (*n* = 5–6 mice per group, scale bar: 200 μm). (**E** and **F**) Histological score of the pancreas and lung tissues from HTG mice with cerulein-induced (**E**) or alcoholic (**F**) pancreatitis scored by 2 pathologists independently (10 images per mouse). (**G**) mRNA level of *Il1b* in the pancreas from HTG mice with cerulein-induced or alcoholic pancreatitis. *Gapdh* was used as an endogenous control. (**H**) Serum IL-6 levels from HTG mice with cerulein-induced or alcoholic pancreatitis. *P < 0.05, **P < 0.01, ***P < 0.001, ****P < 0.0001. Data are presented as mean ± SD and compared by 1-way ANOVA (**A**) or unpaired 2-tailed Student’s *t* test (**B** and **E–H**).

Since the crucial roles of miRNAs have been implicated in various pathological processes, including pancreatitis (26), we performed miRNA sequencing in the pancreas of cerulein-induced AP mice, with or without 4 weeks of administration of P-407. P-407 is an LPL inhibitor that induces similar elevation of TG levels and pathological changes in mice as observed in patients carrying LPL malfunction-associated mutations (5, 27). We found that miR-153 was the most significantly upregulated miRNA in the pancreatic tissue from the P-407–induced HTG group compared with the PBS-treated group (Figure 1A and Supplemental Figure 1, A–C; supplemental material available online with this article; https://doi.org/10.1172/jci.insight.138584DS1). miR-153 upregulation was also observed in the AP tissue of rats with high-fat diet–induced HTG (Supplemental Figure 1D) and plasma of patients with AP with HTG compared with patients and rats without HTG (Figure 1B). Notably, although miR-153 expression was also slightly downregulated in HTG mice with AP stimulation, the extent of downregulation was much smaller compared with non-HTG mice (Figure 1A). From another perspective, pancreatic miR-153 expression in HTG-AP mice was much higher than that in AP mice without HTG. In addition, induction of AP using alcohol plus palmitoleic acid (POA) and bile acid, which are associated with alcoholic and biliary AP, respectively, caused a similar decrease in miR-153 expression as induced by cerulein (Supplemental Figure 1, E and F). Collectively, these data indicate that upregulated miR-153 induced by HTG could be a specific mediator for HTG-associated pancreatitis severity.

To explore the role of pancreatic miR-153 during HTG-AP, we used lentivirus pancreatic in situ injection to induce stable pancreas-specific miR-153 overexpression or miR-153 inhibition in mice (28, 29). The efficiency of lentivirus infection was verified by GFP staining and quantitative PCR (qPCR) quantification of miR-153 (Supplemental Figure 2, A–C). Mice were allowed to recover for 7 days before P-407 injections (Figure 1C). miR-153 overexpression or inhibition alone did not induce any notable changes to the pancreas before AP induction as indicated by H&E staining (Supplemental Figure 2D). Notably, miR-153 did not affect serum TG or FFA levels (Supplemental Figure 2E). Compared with HTG mice injected with control lentivirus, miR-153 inhibition alleviated the severity of both cerulein- and alcohol plus POA–induced pancreatitis with HTG. Acinar cell necrosis and inflammatory cell infiltration were largely reduced by miR-153 inhibition (Figure 1, D–F, and Supplemental Figure 3A). Pancreatic NF-κB activation indicated by phosphorylated p65 and proinflammatory cytokine expression were similarly reduced (Figure 1G and Supplemental Figure 3, B–D). Systemic inflammatory response, as indicated by lung histology and serum IL-6 level, was also alleviated with miR-153 inhibition (Figure 1, D–F, H, and Supplemental Figure 3A). Conversely, miR-153 overexpression in non-HTG mice resulted in significantly increased pancreatic inflammation and necrosis in cerulein-induced pancreatitis (Supplemental Figure 3, E–J). Taken together, these data suggest that upregulated pancreatic miR-153 specifically induced by HTG worsens AP.

### Upregulated miR-153 induced by HTG delays pancreatic repair.

**Figure 2:**
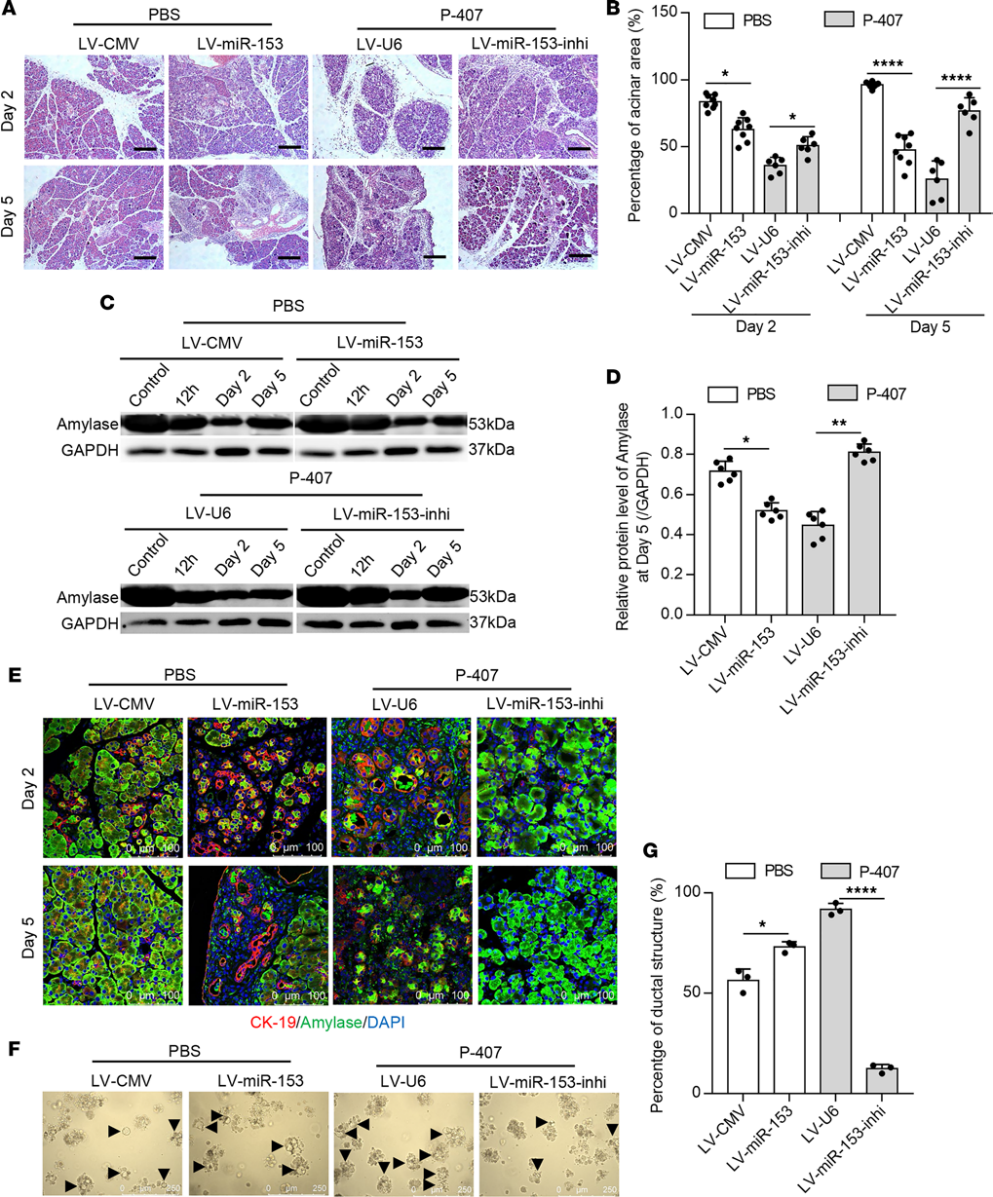
HTG-upregulated miR-153 delays pancreatic regeneration after AP. (**A**) Representative images of H&E-stained pancreas sections at day 2 (top) and day 5 (bottom) after cerulein stimulation (*n* = 6–8 mice per group, scale bar: 200 μm). (**B**) Analysis of the percentage of normal acinar cell area at days 2 and 5 after cerulein stimulation (5 images per mouse). (**C** and **D**) Representative Western blots (**C**) and statistical analysis (**D**) of amylase protein level in pancreas from non-HTG (top) and HTG (bottom) mice at indicated time points after cerulein stimulation. GAPDH was used as an endogenous control. (**E**) Representative images of amylase/CK-19 costained pancreas sections at day 2 (top) and day 5 (bottom) after cerulein stimulation (*n* = 6–8 mice per group, scale bar: 100 μm). (**F** and **G**) Representative images (**F**) and statistical analysis (**G**) of ductal-like structures at day 4 in the 3D culture of primary acinar cells isolated from indicated mice (10 images per group, scale bar: 250 μm). *P < 0.05, ****P < 0.0001. Data are presented as mean ± SD and compared by 1-way ANOVA.

Previous studies have shown that miR-153 could modulate cell stemness and stem cell differentiation (30, 31). Therefore, we sought to determine whether pancreatic miR-153 also mediates tissue repair after AP. A 2-day cerulein injection model was used to stimulate pancreatic regeneration as previously described (32, 33). Persistent miR-153 upregulation was observed throughout the whole pancreatic repair phase in HTG mice (Supplemental Figure 4A). Consistent with a previous report (5), significantly delayed pancreatic regeneration was observed in the HTG group compared with the non-HTG group. miR-153 inhibition in HTG mice significantly reduced tissue edema, inflammatory infiltration, and acinar-to-ductal metaplasia (ADM) structures compared with the control virus-injected group, as indicated by costaining of the ductal cell markers CK-19/SOX-9 and acinar cell marker amylase at days 2 and 5 after cerulein stimulation (Figure 2, A–E, and Supplemental Figure 4B), leading to the rapid restoration of pancreatic exocrine function as assessed by amylase expression (Figure 2, C and D). Correspondingly, miR-153 overexpression in non-HTG mice led to more pancreatic edema, inflammatory infiltration, more severe pancreatic acinus loss, and ADM structure formation at day 2 and failed to achieve a similar degree of recovery by day 5 compared with the control virus-injected group (Figure 2, A–E, and Supplemental Figure 4B). These data suggest that upregulated miR-153 induced by HTG delays tissue repair after AP.

Pancreatic acinar cell proliferation and ADM resolution are 2 main processes to achieve fully pancreatic regeneration (34). We first found that miR-153 did not affect the proliferation of acinar cells as indicated by costaining of amylase and Ki-67 (Supplemental Figure 4, C and D). Because the initial severity of AP could influence ADM formation, we used an in vitro acinar cell 3D culture model to investigate whether miR-153 could independently affect ADM formation. Primary acinar cells were isolated and embedded into collagen Ι as previously described (35). At day 4 of 3D culture, almost all of the primary acinar cells from HTG mice formed ductal-like structures, whereas the number of which was largely reduced by miR-153 inhibition (Figure 2, F and G). Accordingly, miR-153 overexpression promoted the ADM formation in acinar cells from non-HTG mice (Figure 2, F and G). Taken together, these results indicate that HTG-induced miR-153 upregulation delays tissue repair after AP likely via promoting ADM formation.

### miR-153 exacerbates inflammatory responses and acinar-to-ductal metaplasia via targeting TRAF3.

**Figure 3:**
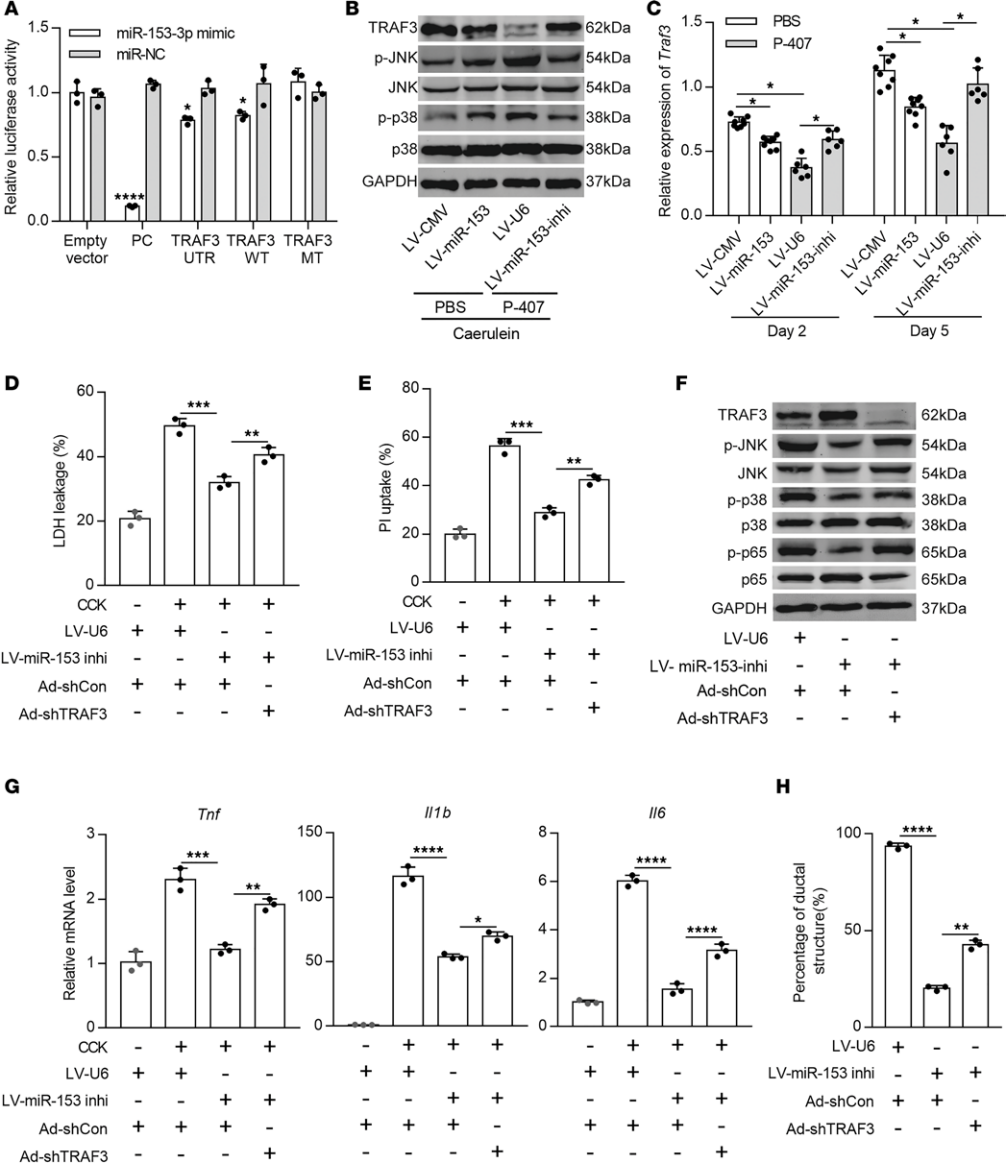
miR-153 promotes inflammatory responses and ADM formation via targeting TRAF3. (**A**) Relative luciferase activity of the luciferase reporter assay. Plasmids carrying the TRAF3 3’-UTR and its wild type (WT) or mutant (MT) counterpart of predicted miR-153 binding site were cotransfected with miR-153-3p mimic or its control to NIH3T3, luciferase activity was measured 48 hours later. (**B**) Representative Western blots showing the expression of TRAF3 and its downstream p38 MAPK/JNK signaling in AP tissues of indicated mice (*n* = 6–8 mice per group). (**C**) qPCR quantification showing relative expression of *Traf3* in the pancreas of indicated mice at days 2 and 5 after cerulein stimulation. *Gapdh* was used as an endogenous control. (**D**) LDH activity in the supernatant of primary acinar cells, an LDH leakage positive control was used to determine percentage of LDH leakage. (**E**) PI uptake by primary acinar cells were measured, total cell counts in each well was determined after Triton X-100 treatment. (**F**) Representative Western blots showing protein level of TRAF3, total and phosphorylated p38, JNK, and p65, in isolated primary acinar cells from 3 independent experiments. (**G**) Relative mRNA level of *Tnf*, *Il1b*, and *Il6* in isolated primary acinar cells. *Gapdh* was used as an endogenous control. (**H**) Statistical analysis of ductal-like structures at day 4 in 3D culture of primary acinar cells (10 images per group). *P < 0.05, **P < 0.01, ***P < 0.001, ****P < 0.0001. Data are presented as mean ± SD and compared by unpaired 2-tailed Student’s *t* test (**A**) or 1-way ANOVA (**C**–**E**, **G**, and **H**).

To explore the downstream targets of miR-153, we used a publicly available database TargetScan (http://www.targetscan.org/) and identified that TRAF3, a scaffold protein that is associated with both acute inflammation (36) and pancreatic regeneration (37), may be a potential functional target of miR-153. We first performed a dual luciferase reporter assay and confirmed the direct interaction between miR-153 and the 3′-UTR of TRAF3 (Figure 3A). We found that TRAF3 expression was downregulated by either direct lentiviral-based miR-153 overexpression or HTG-induced miR-153 overexpression in vivo, which could be restored in HTG with miR-153 inhibition, both during the acute phase (Figure 3B and Supplemental Figure 6, A and B) and the pancreatic regeneration phase (Figure 3C). The activation of the downstream p38/MAPK and JNK signaling, indicated by their corresponding phosphorylated proteins, also changed with miR-153 expression changes (Figure 3B and Supplemental Figure 6, A and B).

To further examine the effect of miR-153-TRAF3 signaling, we isolated pancreatic acinar cells from non-HTG mice with miR-153 overexpression, HTG mice with miR-153 inhibition, and corresponding control mice, then infected the cells with TRAF3 overexpression or knockdown adenovirus and their control adenovirus, respectively. Control adenoviruses did not cause any differences in cell death as indicated by percentage of PI uptake and LDH leakage, with or without cholecystokinin (CCK) stimulation (Supplemental Figure 5, A–F). Altered miR-153/TRAF3 expression was able to slightly influence cell death even without CCK stimulation (Supplemental Figure 5, A–D), the changes of which were much more enlarged after CCK stimulation. Specifically, TRAF3 knockdown partially reversed the protective effect of miR-153 inhibition (Figure 3, D and E), whereas TRAF3 overexpression rescued acinar cells from miR-153 overexpression-induced damages (Supplemental Figure 6, C and D). The activation of proinflammatory signaling p38 MAPK/JNK, indicated by the corresponding phosphorylated proteins and NF-κB and their downstream cytokine expression, was upregulated by TRAF3 knockdown (Figure 3, F and G, and Supplemental Figure 6, E and F), and downregulated by TRAF3 overexpression (Supplemental Figure 6, G–J). Similarly, in vitro 3D culture showed that TRAF3 knockdown promoted ADM formation, whereas TRAF3 overexpression inhibited ADM (Figure 3H and Supplemental Figure 6K). Taken together, these data suggest that upregulated miR-153 induced by HTG aggravates pancreatic inflammation and delays repair likely via suppressing TRAF3.

### SREBP1c transcriptionally inhibits miR-153 to protect against pancreatitis and promotes tissue repair.

**Figure 4:**
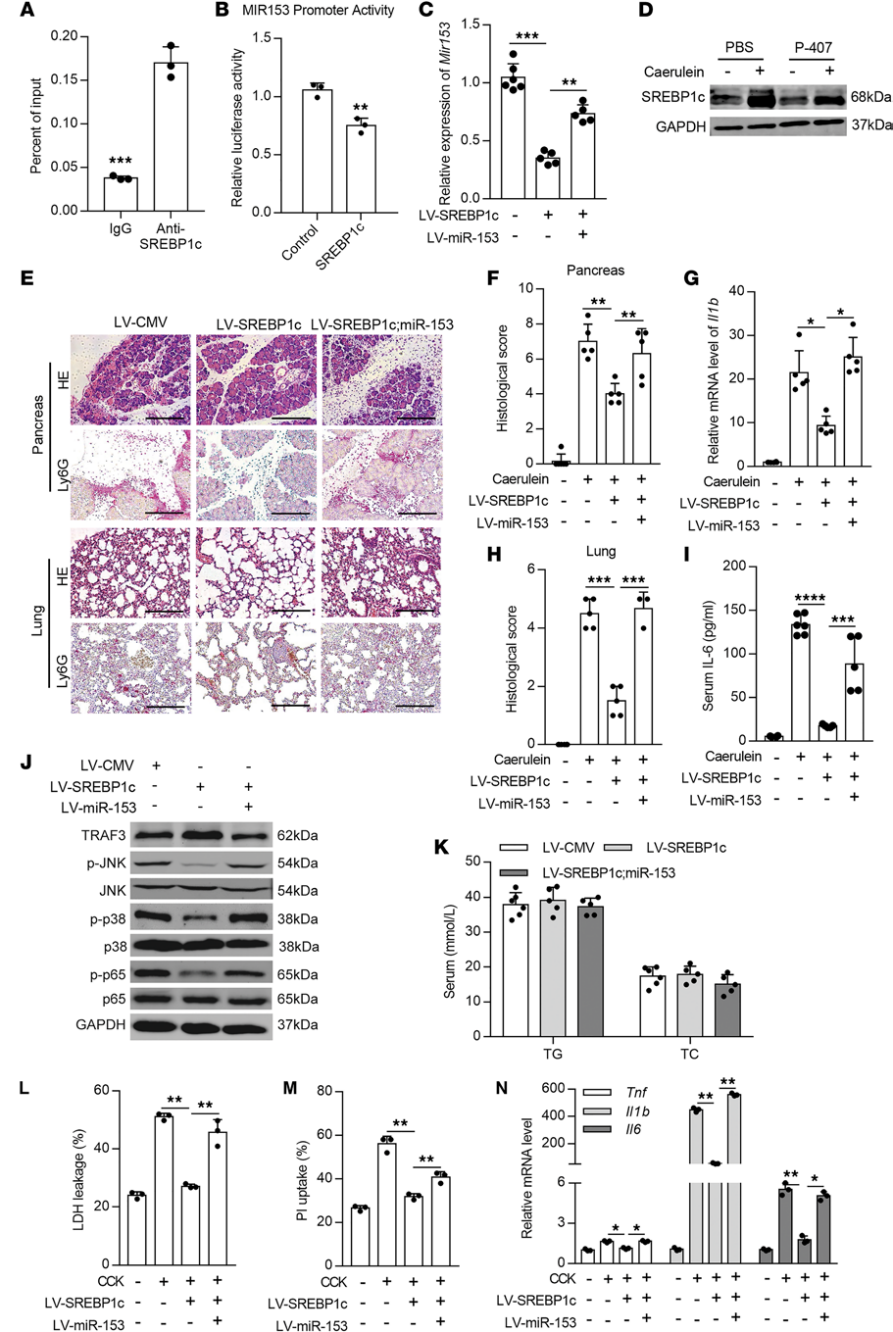
SREBP1c protects mice from HTG-AP through transcriptionally inhibiting miR-153. (**A**) ChIP-qPCR of putative SREBP1c binding sites in Mir153 promoter. (**B**) Relative mRNA expression of *Mir153* in the pancreas. *Rnu6* was used as an endogenous control. (**C**) Dual luciferase reporter assay showing the effect of SREBP1c on Mir153 promoter activity in 293T cell line. (**D**) Representative Western blots showing SREBP1c levels in the pancreas (*n* = 5–6 mice per group). (**E**) Representative images of H&E- and Ly6G-stained pancreas (top) and lung (bottom) sections from HTG-AP mice (*n* = 5–6 mice per group, scale bar: 200μm). (**F**–**H**) Histological score of the pancreas (**F**) and lung (**H**) tissues scored by 2 pathologists independently (10 images per mouse). (**G**) qPCR quantification of *Il1b* in the pancreas. *Gapdh* was used as an endogenous control. (**I**) Serum IL-6 level of HTG mice quantified by ELISA. (**J**) Representative Western blots showing levels of TRAF3, total and phosphorylated p38, JNK, and p65, in the pancreas (*n* = 5–6 mice per group). (**K**) LDH activity in the supernatant of primary acinar cells. (**L**) PI uptake by primary acinar cells. (**M**) qPCR quantification of *Tnf*, *Il1b*, and *Il6* in primary acinar cells, *Gapdh* was used as an endogenous control. *P < 0.05, **P < 0.01, ***P < 0.001, ****P < 0.0001. Data are presented as mean ± SD and compared by unpaired 2-tailed Student’s *t* test (**A** and **B**) or 1-way ANOVA (**C**, **F–I**, and **L–N**).

Since miR-153 expression was upregulated in HTG, we next sought to investigate the upstream regulator of miR-153 expression and focused on the proteins related to TG metabolism. Using in silico analysis, we found a SREBP1c binding site approximately 1300 bp upstream of the miR-153 transcription start site. SREBP1c is the key transcription factor for TG synthesis (38), the role of which in pancreatitis remains to be determined. The putative SREBP1c binding site in miR-153 promoter was first confirmed by ChIP-qPCR in mouse pancreatic tissues (Figure 4A) and further verified using the dual luciferase reporter assay (Figure 4B). The luciferase assay also showed that SREBP1c cotransfection suppressed the activity of miR-153 promoter. Moreover, pancreatic miR-153 expression was significantly suppressed with SREBP1c overexpression mediated by lentivirus injection in the pancreas (Figure 4C), indicating SREBP1c negatively regulates miR-153 transcription. Moreover, we found that SREBP1c expression was upregulated in cerulein-induced AP, but was markedly downregulated in cerulein-induced AP with HTG compared with non-HTG AP (Figure 4D and Supplemental Figure 7A).

We next examined if SREBP1c plays a role in HTG-AP. We established lentivirus-mediated pancreatic SREBP1c overexpression with or without co-overexpression of miR-153. Costaining of GFP and mCherry by immunofluorescence staining indicated successful coexpression of both SREBP1c and miR-153 in the pancreas (Supplemental Figure 7B). SREBP1c and miR-153 expression in the pancreas was confirmed by Western blotting and qPCR, respectively (Figure 4C and Supplemental Figure 7C). In addition, injection of these lentiviruses did not induce any notable changes in the pancreas before AP induction (Supplemental Figure 7D). In HTG mice, local and systemic inflammatory damages were significantly alleviated by SREBP1c overexpression in both cerulein-induced and alcoholic pancreatitis models (Figure 4, E–I, and Supplemental Figure 8, A–C). Co-overexpressing miR-153 with SREBP1c largely reversed the protective effects of SREBP1c. TRAF3 expression was also restored by SREBP1c overexpression in HTG mice, but inhibited by additional miR-153 overexpression (Figure 4J and Supplemental Figure 8D). The p38 MAPK/JNK and NF-κB signaling activation indicated by the corresponding phosphorylated proteins levels and cytokines expression were similarly affected (Figure 4, G and J, and Supplemental Figure 8E). Moreover, serum TG and FFA levels were not affected by SREBP1c overexpression in both AP models (Supplemental Figure 8F). We then isolated primary pancreatic acinar cells from HTG mice preinjected with indicated lentiviruses. Without CCK stimulation, SREBP1c overexpression slightly reduced acinar cell death as assessed by PI uptake and LDH leakage while additional miR-153 overexpression reversed the protective effects (Supplemental Figure 8, G and H). These effects were significantly enhanced after CCK stimulation (Figure 4, K–L). Moreover, proinflammatory cytokine expression in pancreatic acinar cells were similarly affected (Figure 4M). Collectively, these data demonstrate that SREBP1c overexpression alleviates the increased severity of HTG-induced AP via transcriptionally downregulating miR-153.

**Figure 5:**
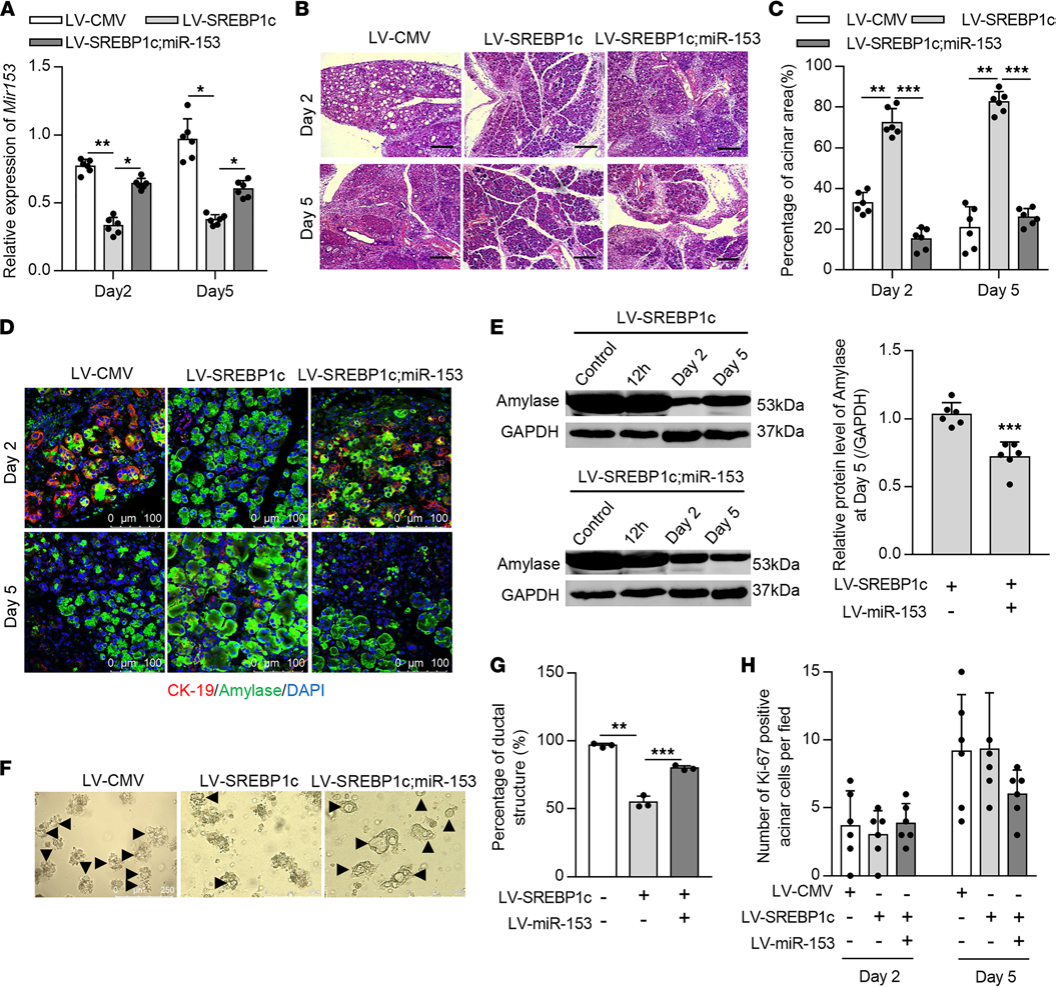
SREBP1c accelerates pancreatic repair in HTG-AP mice via downregulating miR-153. (**A**) qPCR quantification of *Mir153* expression at days 2 and 5 after cerulein stimulation in lentivirus-injected HTG mice. *Rnu6* was used as an endogenous control. (**B**) Representative images of H&E-stained pancreas sections at day 2 (top) and day 5 (bottom) after cerulein stimulation in lentivirus-injected HTG mice (*n* = 6 mice per group, scale bar: 200 μm). (**C**) Analysis of the percentage of normal acinar cell area at days 2 and 5 after cerulein stimulation in the pancreas (5 images per mouse). (**D**) Representative images of amylase/CK-19 costained pancreas sections at day 2 (top) and day 5 (bottom) after cerulein stimulation in lentivirus-injected HTG mice (*n* = 6 mice per group, scale bar: 100 μm). (**E**) Representative Western blots and statistical analysis of amylase protein level at indicated time points after cerulein stimulation in the pancreas. (**F** and **G**) Representative images (**F**) and statistical analysis (**G**) of ductal-like structures at day 4 in 3D culture of primary acinar cells (10 images per group, scale bar: 250 μm). (**H**) Quantification of amylase/Ki-67 double-positive cells in pancreatic sections at days 2 and 5 after cerulein stimulation (10 images per mice). *P < 0.05, **P < 0.01, ***P < 0.001. Data are presented as mean ± SD and compared by 1-way ANOVA.

Next, we explored whether SREBP1c could affect tissue repair after HTG-AP via modulating miR-153. SREBP1c persistently suppressed miR-153 expression during the regeneration process (Figure 5A). SREBP1c overexpression in HTG mice significantly accelerated pancreatic repair as manifested by reduced residual ADM area and increased production of digestive enzymes as assessed by amylase protein level (Figure 5, B–E). Similarly, simultaneous miR-153 overexpression abolished these protective effects of SREBP1c overexpression during pancreatic repair in HTG mice. In vitro 3D culture of primary acinar cells was then performed and revealed that ADM formation was inhibited by SREBP1c but increased by simultaneous miR-153 overexpression (Figure 5, F and G). In addition, SREBP1c did not affect the proliferation of acinar cells as indicated by Ki-67 staining (Figure 5H). Collectively, these data suggest that SREBP1c promotes tissue repair after AP in HTG mice through modulating miR-153.

### Insulin reduces AP severity and promotes tissue repair during HTG-AP via upregulating SREBP1c.

**Figure 6:**
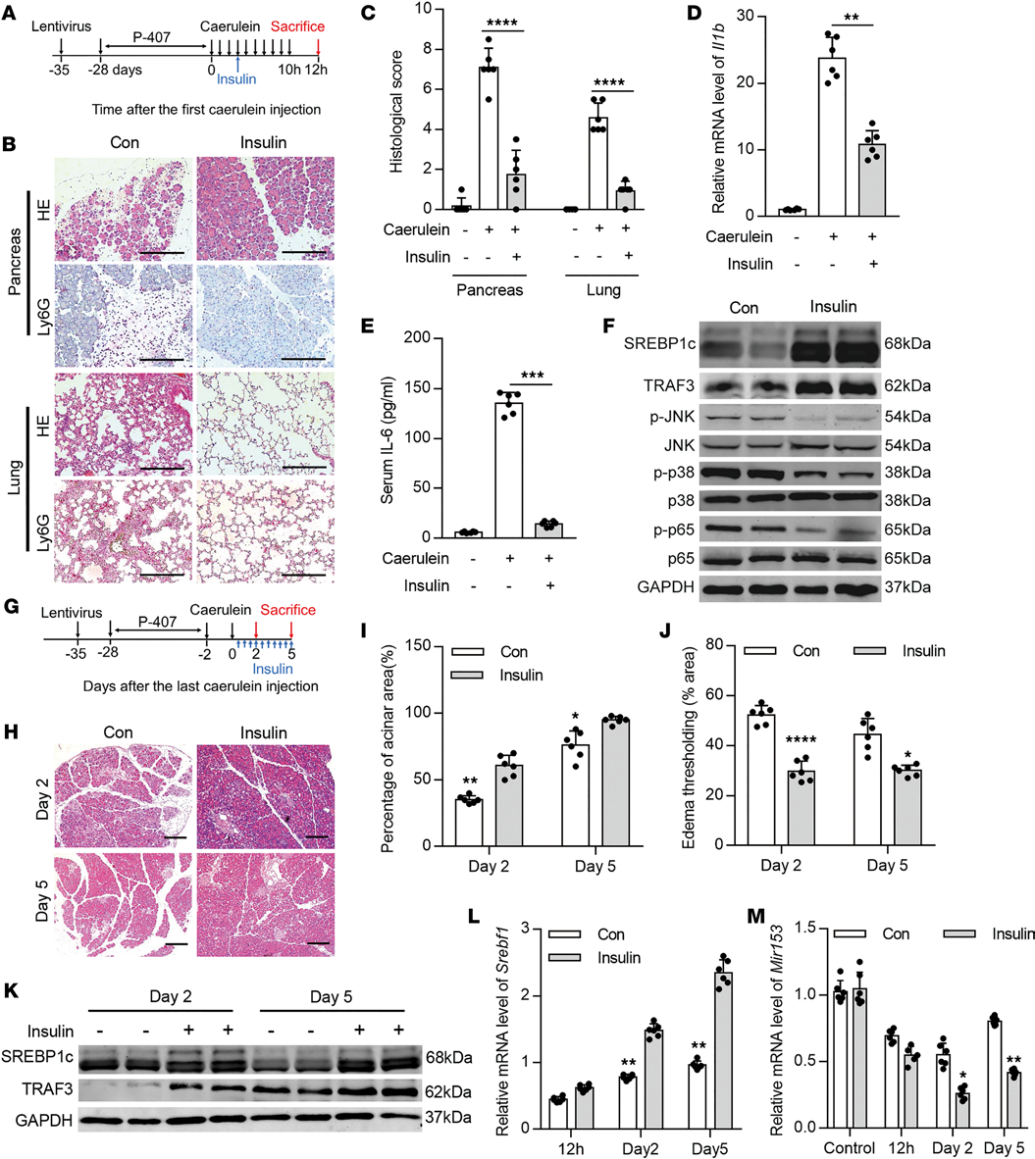
Insulin protects against HTG-AP partially via restoring SREBP1c/miR-153 signaling. (**A**) Schematic of the experimental procedure to imply insulin treatment in HTG-AP. (**B**) Representative images of H&E- and Ly6G-stained pancreas (top) and lung (bottom) sections (*n* = 6 mice per group, scale bar: 200 μm). (**C**) Histological score of the pancreas and lung tissues scored by 2 pathologists independently (10 images analyzed per mouse). (**D**) qPCR quantification of *Il1b* in the pancreas. *Gapdh* was used as an endogenous control. (**E**) Serum IL-6 levels determined by ELISA. (**F**) Representative Western blots showing levels of SREBP1c, TRAF3, total and phosphorylated p38, JNK, and p65 in HTG-AP tissues (*n* = 6 mice per group). (**G**) Schematic of the experimental procedure to imply insulin treatment in the regeneration model. (**H**) Representative images of H&E-stained pancreas sections at day 2 (upper) and day 5 (lower) after cerulein stimulation (*n* = 6 mice per group, scale bar: 200 μm). (**I**) Percentage of normal acinar cell area (5 images per mouse). (**J**) Percentage of edema area quantified by ImageJ (10 images per mouse). (**K**) Representative Western blots showing levels of SREBP1c and TRAF3 in the pancreas (*n* = 6 mice per group). (**L** and **M**) qPCR quantification of *Srebf1* (**L**) and *Mir153* (**M**) expression in the pancreas. *Gapdh* and *Rnu6* were used as an endogenous controls, respectively. *P < 0.05, **P < 0.01, ***P < 0.001, ****P < 0.0001. Data are presented as mean ± SD and compared by unpaired 2-tailed Student’s *t* test.

It is well known that insulin can directly upregulate SREBP1c expression and facilitate SREBP1c maturation (39, 40). Thus, we tested the effect of insulin on AP in LPL malfunction-induced HTG mice. We subcutaneously administered a single dose of insulin (2 IU/kg) in 5% glucose 3 hours after pancreatitis induction (Figure 6A). This dose mimics the clinically used i.v. infusion of insulin at a dose of 0.1–0.3 IU/kg/h. We next monitored blood glucose levels. Insulin administration significantly reduced pancreatic histological damage, necrosis, inflammatory cell infiltration, activation of proinflammatory signals indicated by the corresponding phosphorylated proteins levels, and cytokine expression in HTG-AP (Figure 6, B–D and F and Supplemental Figure 9A). Lung histopathology and inflammatory infiltration were similarly reduced with insulin treatment in HTG-AP (Figure 6, B, C, and E).

Next, we examined whether insulin affected tissue repair after HTG-AP. To rule out the effect of insulin on pancreatitis severity, we subcutaneously applied insulin (2 IU/kg, twice daily) after the last cerulein injection until mice were sacrificed (Figure 6G). We next monitored blood glucose. We found that mice treated with insulin exhibited a trend toward rapid tissue recovery as indicated by increased acinar cell content and markedly decreased ADM area and edema (Figure 6, H–J, and Supplemental Figure 9B). Furthermore, insulin treatment resulted in, as expected, the upregulation of TRAF3 and mature SREBP1c protein levels, and suppression of miR-153 expression in HTG-AP (Figure 6, F, K–M, and Supplemental Figure 9, C–E), indicating that insulin protects against HTG-AP via upregulating SREBP1c. In addition, serum TG and TC levels remained unaffected with insulin treatment during AP but decreased after a few days of insulin treatment (Supplemental Figure 9, F and G). Taken together, these data suggest that insulin reduces disease severity and facilitates tissue repair during HTG-AP partially via restoring SREBP1c. As proof of concept, these data suggest that SREBP1c activator could be a potential therapeutic strategy for treating patients with HTG-AP with LPL malfunction.

## Discussion

Severe and very severe HTG, which are more prone to develop in patents with LPL malfunction, are associated with persistent organ failure and increased mortality in patients with AP (3, 4, 41). However, mechanisms underlying increased AP severity caused by HTG remain elusive. Our study revealed that changes of pancreatic intrinsic molecular signaling SREBP1c/miR-153 governed the natural process of HTG-AP, independent of TG and FFA levels, in P-407–induced HTG mice. We showed that LPL malfunction caused a reduction in pancreatic SREBP1c expression, resulting in the loss of its transcription suppression on miR-153. Increased miR-153 expression by HTG promoted inflammatory damage and acinar cell ADM transformation via targeting TRAF3, thus worsening AP and delaying pancreatic repair. Insulin, an endogenous hormone, showed important therapeutic value for HTG-AP by normalizing the dysregulated SREBP1c/miR-153 signaling.

The critical role of miRNA has been implicated in many diseases, including cancer, metabolic diseases, and AP (42). Using miRNA sequencing, we found that miR-153 was significantly upregulated in different cause-induced HTG in animals and patients with HTG-AP. miR-153 has previously been reported to promote inflammation in cerebral ischemia/reperfusion injury through regulating Nrf2/HO-1 signaling and increasing the production of ROS (43). miR-153 could also influence the development of cancer via modulating stem cell signaling, including Wnt and TGF-β signaling (44, 45). Consistent with these studies, our results demonstrated that increased miR-153 was responsible for the increased severity of AP and impeding of pancreatic repair via targeting TRAF3 in HTG mice. In addition, TRAF3, which negatively regulates MAPK activation and NF-κB signaling (46), has been reported to be involved in regulating inflammatory response in both pancreatic acinar cells (47) and infiltrated macrophages (48) during AP. Collectively, these data suggest that miR-153/TRAF3 could be a critical regulator for HTG-AP.

SREBP1c is a crucial transcriptional regulator of fatty acid and TG synthesis (49). Our study found that it could transcriptionally inhibit miR-153, thus protecting against HTG-AP and facilitating pancreatic repair. Previous studies usually considered SREBP1c as a proinflammatory molecule. Increased expression or functional activation of SREBP1c was closely related to increased proinflammatory responses in several diseases (50, 51). In contrast, our study showed that in P-407–induced HTG mice, overexpression, or activation of pancreatic SREBP1c largely reduced inflammatory response during AP. Although rarely, the protective effect of SREBP1c has also been reported. SREBP1c could prevent lipotoxicity-induced damages via reducing lipid intermediates (52). SREBP1c expression was found to inversely correlate with the severity of inflammation, steatosis, and fibrosis in patients with hepatitis C virus (53). These results indicated the context-dependent role of SREBP1c. Notably, SREBP1c upregulation was found in the pancreas in diet-induced HTG models (54), whereas in LPL malfunction-induced HTG mice, SREBP1c expression was inhibited. However, miR-153 upregulation exists in both HTG models, indicating the existence of other upstream regulators of miR-153 in diet-induced HTG models. Importantly, it implies the existence of distinct mediators for increased AP severity in different HTG etiologies, which emphasizes the importance of specific targeted treatment for patients with AP.

Insulin infusion, which is used as a TG-lowering therapy, has been gradually accepted as a therapeutic option for HTG-AP (4, 25). However, these clinical studies did not distinguish different HTG etiologies, the role of insulin in LPL malfunction-induced HTG-AP, and its underlying mechanisms remain unclear. Our study revealed that insulin could markedly reduce the severity of AP and accelerate tissue repair in LPL malfunction-induced HTG mice via activating SREBP1c. Several reports from in vitro studies indicated that insulin could also alleviate pancreatic acinar cell injury via enhancing plasma membrane Ca^2+^-ATPase activity and glycolysis (55, 56).

In summary, our study addressed the importance of targeting intrinsic signaling changes induced by HTG, more specifically besides lowering TG levels in treating HTG-AP. We identified that dysregulated SREBP1c/miR-153 signaling was responsible for more severe AP and impaired pancreatic repair in HTG mice. Targeting SREBP1c using insulin successfully protects against HTG-AP. These findings shed light on specific treatment of patients with AP and on potential targets for the treatment of LPL malfunction-induced HTG-AP.

## Methods

For information on plasma RNA extraction and quantification, plasmids construction and lentivirus packaging, primary acinar cell isolation, proteins and RNAs quantification, H&E staining and immunostaining, and other in vitro experiment details, see Supplemental Methods.

### Human samples.

Patients diagnosed with AP (57), who were admitted to the Department of Gastroenterology or Department of Emergency, Shanghai General Hospital, from May 2018 to January 2019, were recruited. Patients with AP with other inflammatory diseases were excluded. Patient-informed consent was obtained according to the study protocol, which was approved by the ethics committee of Shanghai General Hospital (2017KY170). Blood was collected within 24 hours after admission, using EDTA as anticoagulant, and centrifuged at 1750*g* for 15 minutes to obtain plasma within 6 hours. The plasma was then stored at –80°C until further analysis. HTG was defined as serum TG level (≥1.7 mmol/L).

### Animal models.

C57BL/6 male mice (6–10 weeks) and Sprague-Dawley rats weighing 120–150 g were purchased from Shanghai SLAC Laboratory Animal Co Ltd, and all experimental procedures were approved by the Animal Ethics Committee of Shanghai General Hospital (2019-A053-01). The HTG mouse model was induced by i.p. injection of P-407 (Sigma-Aldrich, P2443) every other day for 4 weeks. Mice with equal volume of PBS injection served as the control. For high-fat diet–induced HTG, rats were fed with high-fat diet (77% normal chow + 20% saturated animal fat, lard + 3% cholesterol) or normal chow for 4 weeks. AP in rat was induced by 2 hourly i.p. injections of cerulein (50 μg/kg; MCE, HY-A0190), and humane killing was performed 12 hours after the first injection. Mice with equal volume of saline injection served as the control. Cerulein-induced AP in mice was induced by 10 hourly i.p. injections of caerulein (100 μg/kg), humane killing was performed 12 hours after the first injection. Mice with equal volume of saline injection served as the control. Alcoholic AP was induced by 2 hourly i.p. injections of ethanol (1.35 g/kg) and palmitoleic acid (150 mg/kg) as previously reported (58, 59), and humane killing was performed 24 hours later. Mice with equal volume of ethanol injection served as the control. Pancreatic regeneration mouse model was induced by 8 hourly i.p. injections of caerulein (100 mg/kg) per day for 2 days, and mice were sacrificed at indicated time points. Insulin was administered subcutaneously at a dose of 2 IU/kg in 500 μL 5% glucose. Additional 100 μL 10% glucose were supplied through oral gavage when blood glucose level was lower than 3.9 mmol/L. Mice with equal volume of 5% glucose injection served as the control. No specific randomization was used to determine how animals were allocated to experimental groups, and no blinding was done.

### Lentivirus injection.

For in situ injection, mice were anesthetized using 1% pentobarbital sodium (50 mg/kg). A 2 cm incision on the upper left side of the abdomen was made, and the tail of the pancreas along with spleen were exposed. Multiple sites at the tail of the pancreas were injected with lentiviruses at a concentration of at least 5 × 10^9^ IFU/mL (100 μL, at least 5 × 10^8^ IFU lentiviruses in total). Mice were recovered at 37°C until fully awake. Seven days after surgery, different models were further induced.

### miRNA sequencing.

High-throughput sequencing of miRNAs was performed by CloudSeq Biotech. Briefly, the quantity and purity of total RNAs were determined using the NanoDrop ND-100 (Thermo Fisher). Total RNA samples were used to prepare the miRNA sequencing libraries, and approximately 150 bp PCR amplicons (corresponding to approximately 22 nt miRNAs) were selected. The libraries were then applied for RNA sequencing with a HiSeq sequencer (Illumina) by routine. The results were uploaded to the GEO database (GSE138745).

### Statistics.

All experiments were repeated at least 3 times. Data are presented as mean ± SD. Unpaired 2-tailed Student’s *t* test was used for comparisons between 2 groups. Comparisons between more than 2 groups were performed using 1-way ANOVA. All statistical analyses were performed using GraphPad Prism 7.0 software. A *P* value of less than 0.05 was considered significant.

### Study approval.

All studies involving human samples were approved by the ethics committee of Shanghai General Hospital (2017KY170). All animal procedures and experiments were approved by the Animal Ethics Committee of Shanghai General Hospital (2019-A053-01).

## Author contributions

JD, GH, and LW designed the study. JD, MJ analyzed the data. JD, MJ, YH, and LW performed the experiments and wrote the manuscript. J. Xiao, BH, J. Xu, XH, SS, BL, ZW, YH and YR performed the experiments or analyzed the data. LW, XW, and GH supervised the project and revised the manuscript. LW, XW, and GH provided funding to support the study. The degree of contribution to the design, to performance of the experiments, and to manuscript writing were used to assign authorship order among co–first authors.

## Supplementary Material

Supplemental data

## Version 1 01/25/2021

Electronic publication
