# Supplementary material for: Dysregulated SREBP1c/miR-153 signaling induced by hypertriglyceridemia worsens acute pancreatitis and delays tissue repair
Source: JCI Insight. 2021 Jan 25;6(2):e138584. doi: 10.1172/jci.insight.138584 (PMC7934861; doi:10.1172/jci.insight.138584)
Supplement: Supplemental data [file jciinsight-6-138584-s227.pdf]

Supplementary Materials

Supplementary materials contain Supplementary materials and methods, nine Supplementary Figures, and one Supplementary Table.

Supplementary Methods

Plasma RNA extraction and quantification: RNA was extracted from 200 ul plasma of each patient using miRNeasy Serum/Plasma Kit (Qiagen) according to the manufacturer's protocol. Ce_miR-39_1 was used as spike in control (Qiagen). The extracted RNA was then reverse transcribed to DNA using miScript II RT Kit (Qiagen). miScript SYBR Green PCR Kit (Qiagen) was used for further RNA quantification. Primers of miR-153-3p and spike in control were all purchased from Qiagen.

Plasmid construction: All backbone lentivirus plasmids were purchased from OBiO Technology Corporation (Shanghai, China). The *Mir153* gene with flanking sequences was amplified from mouse genome using forward primer 5'-CGGGATCCCAGGCACCCAAGATGTGTAA-3' and reverse primer 5'-CTAGCTAGCGTGAGTCGCCATGCAAACGA-3'. The amplified PCR product and plenti-GFP (lentivirus plasmid expressing a CMV promoter and GFP protein) were double digested with BamHI/NheI (NEB), and then ligated with T4 ligase (NEB). The miR-153-3p inhibitor sequence was designed according to the tough decoy construct(1) and synthesized by Sangon Biotech Corporation (Shanghai, China). The sequence of miR-153 inhibitor is: 5'-GGACTAGTGGCGCTAGGATCATCAACTTGCATAGTCA ATCTCAAAAGTGATCCAAGTATTCTGGTCACAGAATACAACTTGCATAGTCA

ATCTCAAAAGTGATCCAAGATGATCCTAGCGCCACCTTTTTGCTAGCTAG-3'. Sequence of the control scramble: 5'-CCTAAGGTTAAGTCGCCCTCGCTC-3'(2). After double digested with SpeI/NheI (NEB), the miR-153-3p inhibitor was cloned into the plv-GFP plasmid (lentivirus plasmid expressing a U6 promoter and GFP protein). The recombinant plasmid was referred to as plv-miR-153-inhi. The *Srebf1* overexpression plasmid was constructed by the OBiO Technology Corporation (Shanghai, China) using plenti-mCherry.

The 3'-untranslated region (UTR) region of *Traf3* (621bp) was amplified from mouse genome using forward primer 5'-CGAGCTCGTGTGAGCAGAAGCCACA-3' and reverse primer 5'-GCTCTAGAAACGCTTCAGCCATGGAAG-3'. The putative counterparts (wild- and mutant-type) of miR-153 target sequence in the 3'UTR of *Traf3* and a positive control of miR-153 target sequence were synthesized by Sangon biotech. After double digested with SacI/XbaI, the 3'UTR, wild-type (WT), mutant-type (MT) and positive control (PC) segments were cloned into the corresponding digested plasmid pmirGLO vector (7350bp, Promega).

The promoter of miR-153 was amplified from mouse genome using forward primer 5'-CGAGTCCAGCTGCCCTGCAGATTCTC-3' and reverse primer 5'-GCTCTGTTTCTATCTCAACACTCTCCTT-3'. After double digested with SacI/XbaI, the DNA segment was cloned into the corresponding digested plasmid pmirGLO vector.

Lentivirus packaging: pMD2.G (Addgene #12259), psPAX2 (Addgene #12260) were gifts from Didier Trono and used as packaging and envelope plasmids and transfected with the corresponding plasmids to 293T cells using lipofectamine 3000 (Invitrogen)

according to the manufacturer's protocol. The cell supernatant was collected 48 and 96 hours after transfection. The 293T cells were collected 96 hours after transfection. Cells were repeatedly frozen and thawing 3 times, and centrifuged at 10000 rpm for 30 minutes to collect supernatant. Then the collected supernatant was centrifuged at 62500 g for 90 minutes to collect lentivirus particles. The collected lentiviruses were resuspended in DME/F-12 medium and quantified using both 293T cell infection and Lenti-X™ qRT-PCR Titration Kit (Takara).

Serological test: Blood samples from mice were centrifuged at 3000 rpm for 15 minutes at 4°C to collect the serum. Serum amylase, TG and TC levels were measured using the automated biochemical analyzer (Advia 1650, Bayer, Germany). Serum cytokines, including IL-1β, IL-6 and TNF-α were measured using Enzyme-Linked Immunosorbent Assay (ELISA) kits (Multi Sciences, #70-EK201BHS, 70-EK206HS, 70-EK282HS) according to the manufacturer's instructions.

H&E staining, immunohistochemistry, and immunofluorescence: Fresh tissues were fixed with 4% neutral paraformaldehyde at room temperature for 12 to 24 hours, embedded in paraffin and processed into 4 μm sections. H&E staining was done as previously described(3). Antigen retrieval of immunohistochemistry (IHC) staining for Ly6G was achieved by 10 minutes digestion with proteinase K (20 μg/ml, Beyotime, #ST532). Sections was then incubated with Ly6G antibody at 1:100 (Abcam, #ab25377) overnight at 4°C and then incubated with alkaline phosphatase-linked goat-anti-rat secondary antibody (Abcam, #ab6846). SIGMA FAST™ Fast Red TR/Naphthol AS-MX Tablets (Sigma-Aldrich, #F4648) was used for the detection.

Antigen retrieval of immunofluorescence staining was achieved with heat in citrate antigen retrieval solution (Sangon, #E673002). After antigen blocking, primary antibodies were incubated overnight 4°C. The primary antibodies were GFP (Abcam, #ab6673) at 1:200, mCherry (Abcam, #ab25096) at 1:200. After secondary antibody incubation, DAPI was used for nucleus staining. Sections were imaged with confocal microscope (LEICA, SP8).

Cell line: 293T (ATCC® CRL-3216™) and NIH/3T3 (ATCC® CRL-1658™) cells were purchased from the Type Culture Collection of the Chinese Academy of Sciences, Shanghai, China. Both cell lines were cultured in DMEM medium (HyClone) supplemented with 10% FBS (Gibco) at 37°C in a humid atmosphere with 5% CO_2.

Primary acinar cell isolation and culture: Primary mouse pancreatic acinar cells were isolated as previously described(4). In brief, the pancreas tissue was injected with 0.2 mg/ml collagenase IV (Sigma-Aldrich) and digested for 15-20 minutes at 37°C. Tissues were then transferred into wash buffer, which is HBSS supplied with 5% BSA (Sigma-Aldrich). Acinar cells were isolated by pipetting with blunted pipette tips. After filtrating through a 70 µm filter, cells were washed twice in wash buffer and resuspend in culture DME/F-12 medium containing 0.1mg/ml trypsin inhibitor (Sigma-Aldrich), 10% FBS (Gibco), penicillin (75 µg/mL) and streptomycin (100 µg/mL).

Adenovirus infection: Adenoviruses were purchased from OBiO Technology Corporation (Shanghai, China), including one *Traf3* overexpression adenovirus, 3 *Traf3* knockdown adenoviruses using shRNA and their control viruses, respectively. After isolation, primary pancreatic acinar cells were immediately infected with 10^7 PFU/ml

adenovirus for 5 hours, and resuspend in fresh culture medium and analyzed 16 h after.

Propidium iodide (PI) uptake: PI uptake was used to measure cell necrosis, as previously described(5). Briefly, primary pancreatic acinar cells after treatment were plated into 96-well plate, stained with 25 µg/mL PI (Sigma-Aldrich, #P4170) for 5 minutes. Fluorescence was measured at 536 nm excitation and 617 nm emission wavelengths. Fluorescence representing total number of cells for normalization was also measured after lysing cells with 0.5% Triton-X 100.

LDH leakage: LDH leakage was determined using an LDH Release Assay Kit (Beyotime, C0016) according to the manufacturer's protocol. Supernatants of primary pancreatic acinar cells were collected, plated into 96-well plate and incubated with INT solution at room temperature for 30 minutes. Absorbance was detected at 490 nm. A positive control was set in every experiment, where LDH in all cells were released by an LDH releasing regent.

Chromatin immunoprecipitation (ChIP)-qPCR: Fresh harvested pancreatitis tissue was minced and immediately cross-linked using formaldehyde. Tissue was grinded using medimachine from BD Biosciences according to manufacturer's instructions. The fully grinded tissue was digested by micrococcal nuclease and then sonicated for 20 seconds 3 times. The digested, cross-linked chromatin was incubated with either anti-SREBP1 antibody (Santa cruz) or IgG (CST) overnight at 4°C with rotation. Further ChIP procedures were processed according to the manufacturer's protocol (CST, #9004). Forward primer 5'- TCCAGCTGCCCTGCAGATTCTC-3' and reverse primer 5'- TGTTTCTATCTCAACACTCTCCTT-3' were used for further PCR quantification.

Dual luciferase reporter assay: The Dual luciferase reporter assay was performed to verify the target site of miR-153 in TRAF3 and the transcriptional regulation of SREBP1c on miR-153. For verification the target site of miR-153 in TRAF3, 293T cells were co-transfected with pmirGLO plasmids containing miR-153 target sites or 3'UTR of TRAF3 as previous described and miR-153 mimic or mimic NC. For verification the transcriptional regulation of SREBP1c on miR-153, 293T cells were co-transfected with pmirGLO plasmids containing miR-153 promoter region and SREBP1c-overexpressing plasmid as previously described. Cells were collected 48 hrs later and subjected to analyze the dual luciferase activities using Dual-Luciferase® Reporter Assay System (Promega) following the manufacturer's protocol. The dual luciferase activities were measured by Varioskan Flash (Thermo Scientific). Luciferase activities were calculated by the ratio of the luminescence of firefly to renilla, and normalized to control.

Tissue RNA extraction and RT-qPCR: Total RNA was extracted from tissue with TRIzol Reagent (TakaRa). Briefly, total RNA was separated by chloroform and further precipitated by isopropanol. After washed by 75% ethanol, total RNA was dissolved in RNase-free water. RNA was then reverse transcribed as cDNA using PrimeScriptTM RT reagent kit (TakaRa) according to the manufacturer's protocol. SYBR® Premix Ex TaqTM (TakaRa) was used for further real-time PCR. qPCR was performed on the applied biosystems (Life technologies). The relative gene expression was analyzed using the $2^{-\Delta\Delta CT}$ method. *Gapdh* and *Rnu6* were used as endogenous controls, respectively. The primers used in this study are listed in **Supplementary Table 1**.

Western blot: The proteins were extracted from mouse tissue with RIPA lysis buffer (Thermos Fisher) supplied with protease inhibitor cocktail tablets (Roche). Protein samples were separated by SDS-PAGE and transferred onto nitrocellulose filter membranes (Millipore, USA). Membranes were incubated with the primary antibodies overnight at 4°C. The following primary antibodies were used: SREBP1 at 1:500 (Santa Cruz, #sc-13551), TRAF3 at 1:200 (CST, #4729), p65 at 1:1000 (CST, #6956), phosphor-p65 at 1:1000 (CST, #3033), p38 at 1:1000 (CST, #8690), phospho-p38 at 1:1000 (CST, #4511), JNK at 1:1000 (CST, #9252), phospho-JNK at 1:1000 (CST, #4668), amylase at 1:1000 (Santa Cruz, #sc-46657), CK-19 at 1:500 (Abcam, #ab52625), GAPDH at 1:1000 (CST, #2118). The membranes were then probed with IR-Dye 680RD or 800CW labelled secondary antibody for 1 hour at room temperature, and imaged using Odyssey CLx scanner (LI-COR, Lincoln, NE, USA).

Reference:

1. Yoo J, Hajjar RJ, and Jeong D. Generation of Efficient miRNA Inhibitors Using Tough Decoy Constructs. *Methods Mol Biol.* 2017;1521(41-53.
2. Sarbassov DD, Guertin DA, Ali SM, and Sabatini DM. Phosphorylation and regulation of Akt/PKB by the rictor-mTOR complex. *Science.* 2005;307(5712):1098-101.
3. Zhang XX, Deng LH, Chen WW, Shi N, Jin T, Lin ZQ, Ma Y, Jiang K, Yang XN, and Xia Q. Circulating microRNA 216 as a Marker for the Early Identification of Severe Acute Pancreatitis. *Am J Med Sci.* 2017;353(2):178-86.
4. Wen L, Voronina S, Javed MA, Awais M, Szatmary P, Latawiec D, Chvanov M, Collier D, Huang W, Barrett J, et al. Inhibitors of ORAI1 Prevent Cytosolic Calcium-Associated Injury of Human Pancreatic Acinar Cells and Acute Pancreatitis in 3 Mouse Models. *Gastroenterology.* 2015;149(2):481-92 e7.
5. Jin S, Orabi AI, Le T, Javed TA, Sah S, Eisses JF, Bottino R, Molkentin JD, and Husain SZ. Exposure to Radiocontrast Agents Induces Pancreatic Inflammation by Activation of Nuclear Factor-kappaB, Calcium Signaling, and Calcineurin. *Gastroenterology.* 2015;149(3):753-64 e11.

Supplementary Figures

Supplementary figure 1

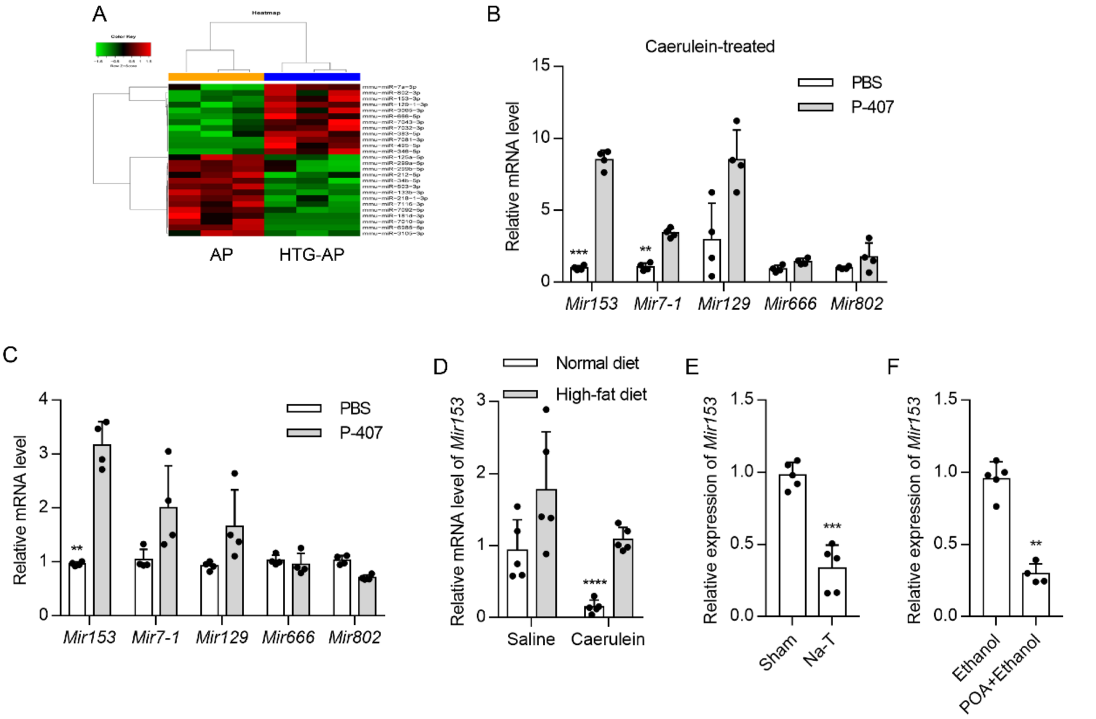

Figure. S1 miR-153 is upregulated in HTG-AP models. A Heatmap of miRNA sequencing comparing the pancreas tissue from caerulein-induced AP with/without P-407 induced HTG. **B, C** qPCR quantification of top 5 upregulated miRNAs in HTG mice stimulated by caerulein (**B**) or saline (**C**) compared to non-HTG mice. *Rnu6* was used as an endogenous control. **D** qPCR quantification of *Mir153* in the pancreas of rats fed with high-fat diet or normal diet. *Rnu6* was used as an endogenous control. **E, F** qPCR quantification of *Mir153* in the pancreas of bile acid (**E**) and alcohol (**F**) induced AP mice. *Rnu6* was used as an endogenous control. n = 4-6 mice per group. **P < 0.01, ***P < 0.001. Data are presented as Mean±SD. (Student's t-test).

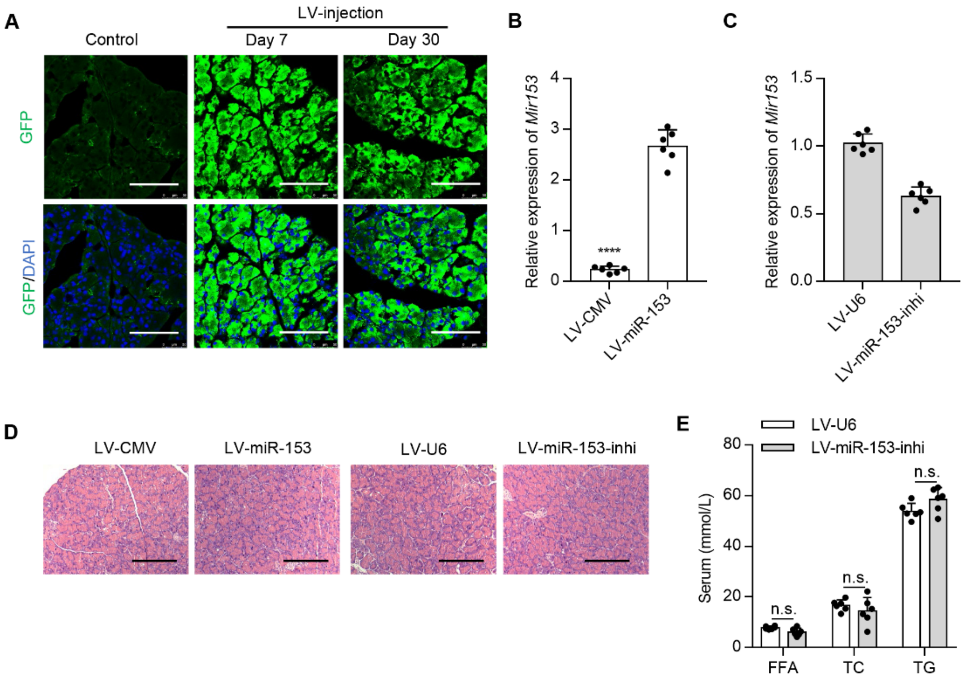

Figure. S2 Lentivirus in situ injection mediates pancreas-specific miR-153 expression *in vivo*. **A** Representative image of GFP (green) staining in the pancreas 30 days after in situ lentivirus injection. Nuclei were labeled with DAPI (blue) (n = 6 mice per group, scale bar=50 μm). **B, C** qPCR quantification of *Mir153* expression in the pancreas of miR-153 overexpressed (**B**) and inhibited (**C**) mice. *Rnu6* was used as an endogenous control. **D** Representative images of H&E-stained pancreas sections of in situ lentivirus-injected mice (n = 5-6 mice per group, scale bar = 200μm). **E** Serum FFA, TC and TG levels in HTG mice. ****P < 0.0001. Data are presented as Mean±SD. (Student's t-test).

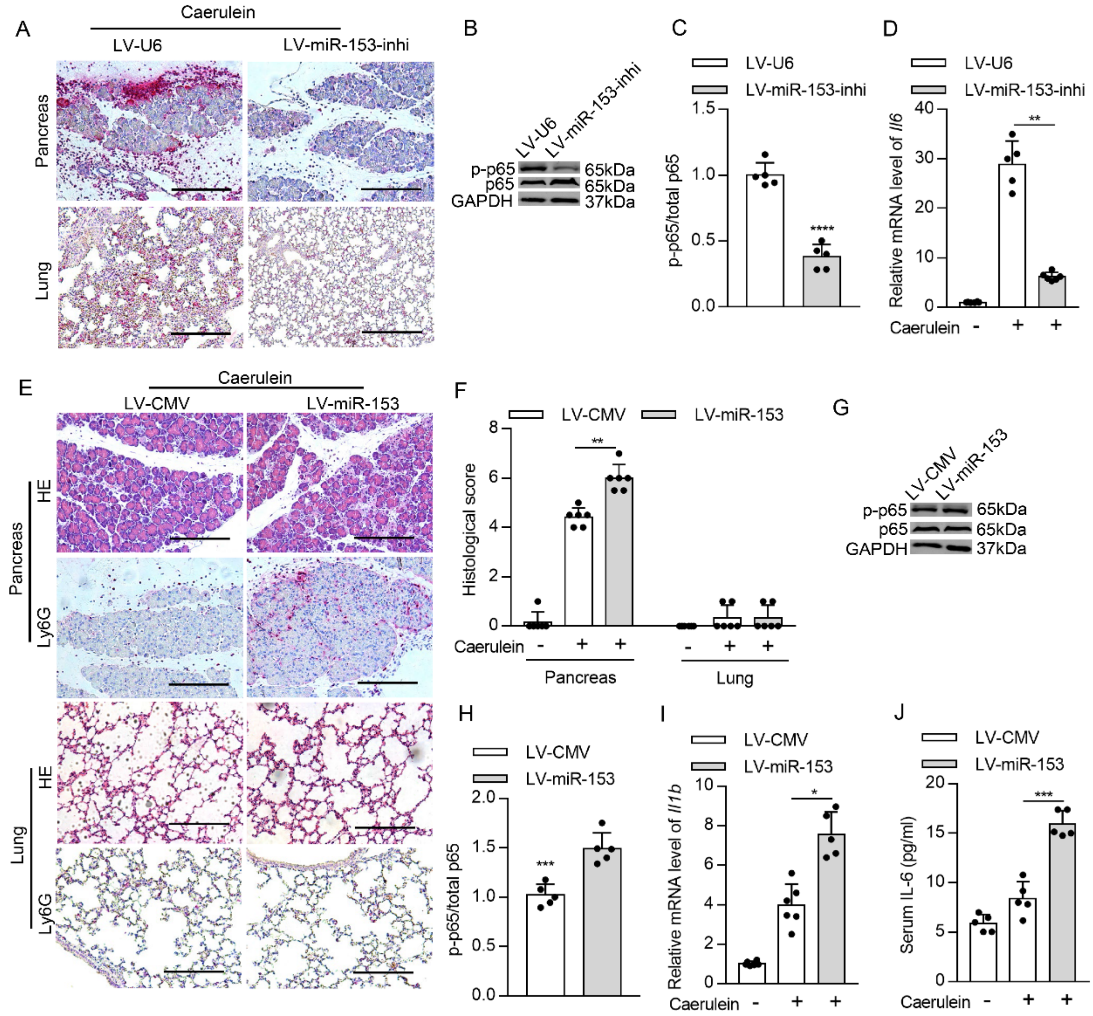

Figure. S3 miR-153 aggravates AP. A Representative images of Ly6G-stained pancreas and lung sections of *in situ* lentivirus-injected HTG mice with caerulein-induced AP (n = 5-6 mice per group, scale bar = 200μm). **B, C** Representative western blots (**B**) and statistical analysis (**C**) showing levels of total and phosphorylated p65 in the pancreas of in situ lentivirus-injected HTG mice with caerulein-induced AP. **D** mRNA level of *Il6* in the pancreas of HTG mice. *Gapdh* was used as an endogenous control. **E** Representative images of H&E and Ly6G-stained pancreas (upper) and lung (lower) sections from *in situ* lentivirus-injected non-HTG mice (n = 5-6 mice per group, scale bar = 200μm). **F** Histological score of the pancreas and lung tissues scored by two pathologists independently (10 images per mouse). **G, H** Representative western blots (**G**) and statistical analysis (**H**) showing levels of total and phosphorylated p65 in the pancreas of *in situ* lentivirus-injected non-HTG mice with caerulein-induced AP. **I** mRNA level of *Il1b* in the pancreas. *Gapdh* was used as an endogenous control. **J** Serum IL-6 levels of non-HTG mice. *P < 0.05, **P < 0.01, ***P < 0.001, n.s.: not significant. Data are presented as Mean±SD. (In (C, H), Student's t-test; In (D, F, I, J), One-way ANOVA).

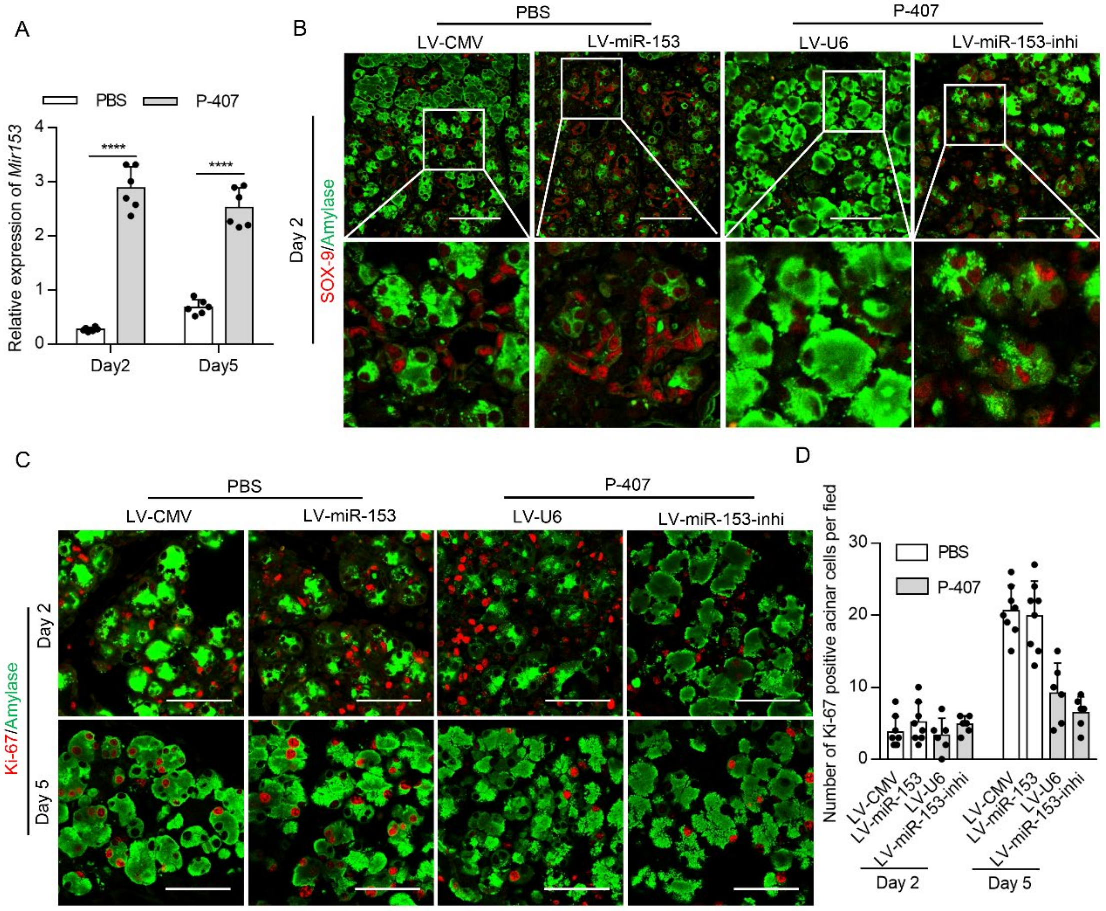

Figure. S4 miR-153 impairs pancreatic recovery after HTG-AP. A qPCR quantification of *Mir153* expression in the pancreas Day 2 and Day 5 after caerulein stimulation. *Rnu6* was used as an endogenous control. **B** Representative images of co-staining of amylase (green) and SOX-9 (red) from the pancreas of *in situ* lentivirus-injected mice at Day 2 after caerulein stimulation. Nuclei were labeled with DAPI (blue). (*n* = 6 mice per group, Scale bar = 50 μm) **C, D** Representative images (**C**) and statistical analysis (**D**) of co-staining of amylase (green) and Ki-67 (red) at Day 2 and Day 5 after caerulein stimulation. Nuclei were labeled with DAPI (blue). Scale bar = 50 μm. ****P < 0.0001. Data are presented as Mean±SD. (Student's t-test).

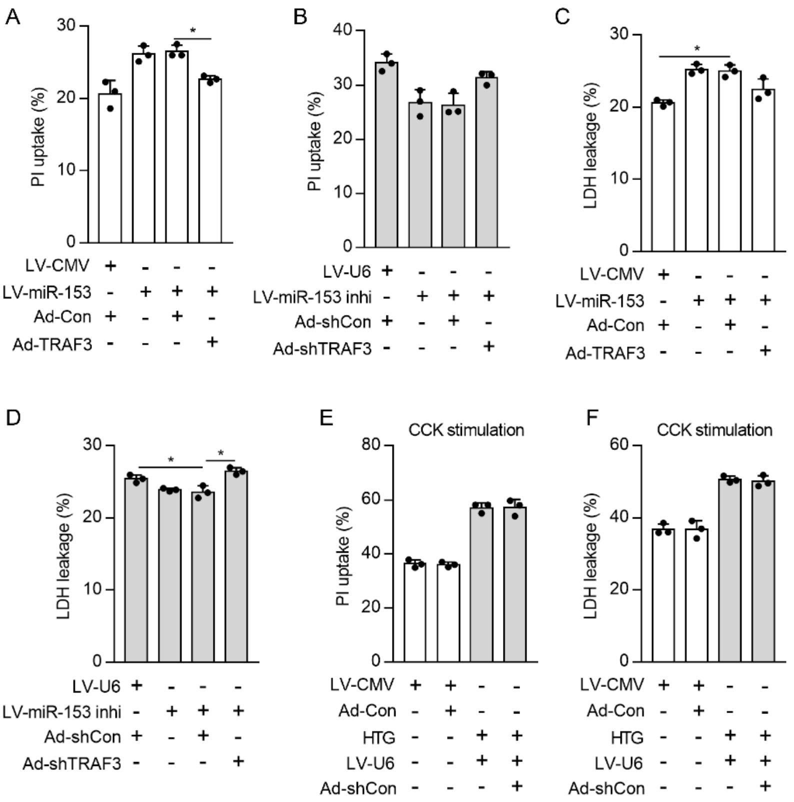

Figure. S5 Control viruses didn't affect acinar cell death. A, B PI uptake were measured in pancreatic acinar cells infected with indicated adenovirus after isolated from non-HTG (**A**) and HTG (**B**) mice in situ-injected with indicated lentivirus, total cell counts in each well was determined after TritonX-100 treatment. **C, D** LDH activity in the supernatant of primary acinar cells infected with indicated adenovirus after isolated from non-HTG (**C**) and HTG (**D**) mice in situ-injected with indicated lentivirus was measured, an LDH leakage positive control was used to determine percentage of LDH leakage. **E** PI uptake were measured in pancreatic acinar cells after CCK stimulation, total cell counts in each well was determined after TritonX-100 treatment. **F** LDH activity in the supernatant of primary acinar cells after CCK stimulation, an LDH leakage positive control was used to determine percentage of LDH leakage. *$P < 0.05$. Data are presented as Mean ±SD. (One-way ANOVA).

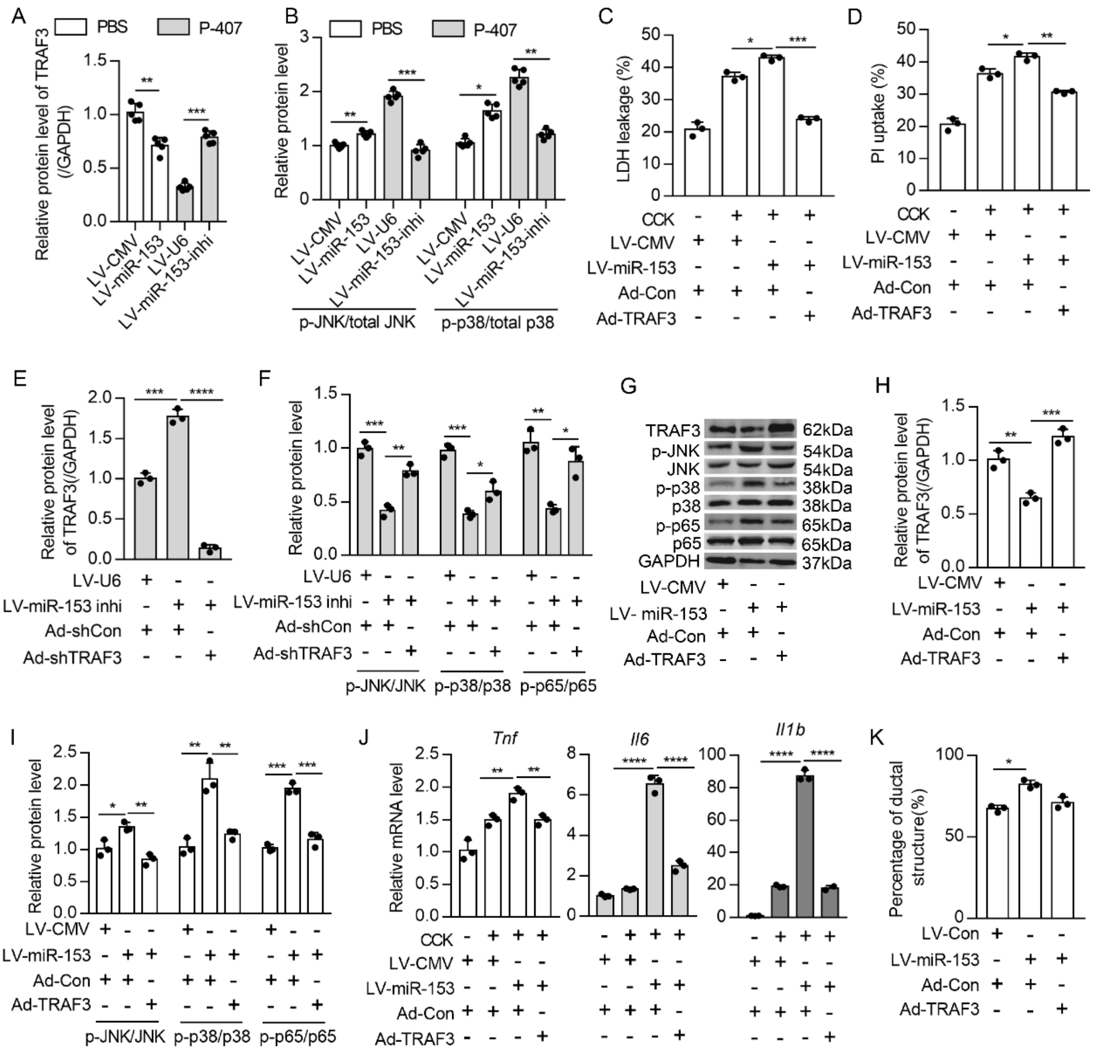

Figure S6 miR-153 increased inflammatory responses and ADM in primary pancreatic acinar cells. A Statistical quantification of TRAF3 expression in lentivirus-injected pancreas from figure 3B. **B** Statistical analysis showing relative p-JNK and p-p38 expression normalized by total JNK and p38 respectively in lentivirus-injected pancreas from figure 3B. **C** PI uptake were measured in pancreatic acinar cells infected with indicated adenovirus after isolated from non-HTG mice, total cell counts in each well was determined after TritonX-100 treatment. **D** LDH activity in the supernatant of primary acinar cells infected with indicated adenovirus after isolated from non-HTG mice *in situ*-injected with indicated lentivirus was measured, an LDH leakage positive control was used to determine percentage of LDH leakage. **E** Statistical quantification of TRAF3 expression in lentivirus-injected pancreas from figure 3F. **F** Statistical analysis showing relative p-JNK, p-p38 and p-p65 expression normalized by total JNK, p38 and p65 respectively in lentivirus-injected pancreas from figure 3F. **G-I** Representative western blots **(G)** and quantification **(H, I)** showing the expression of TRAF3 and its downstream p38 MAPK/JNK signaling in pancreatic acinar cells infected with indicated adenovirus after isolated from non-HTG mice. **J** mRNA levels of *Tnf*, *Il1b*, and *Il6* in isolated primary acinar cells, *Gapdh* was used as an endogenous control. **K** Statistical analysis of ductal-like structures at Day 4 of 3D culture (10 images per group). $^*P < 0.05$, $^{**}P < 0.01$, $^{***}P < 0.001$, $^{****}P < 0.0001$. Data are presented as Mean±SD. (One-way ANOVA).

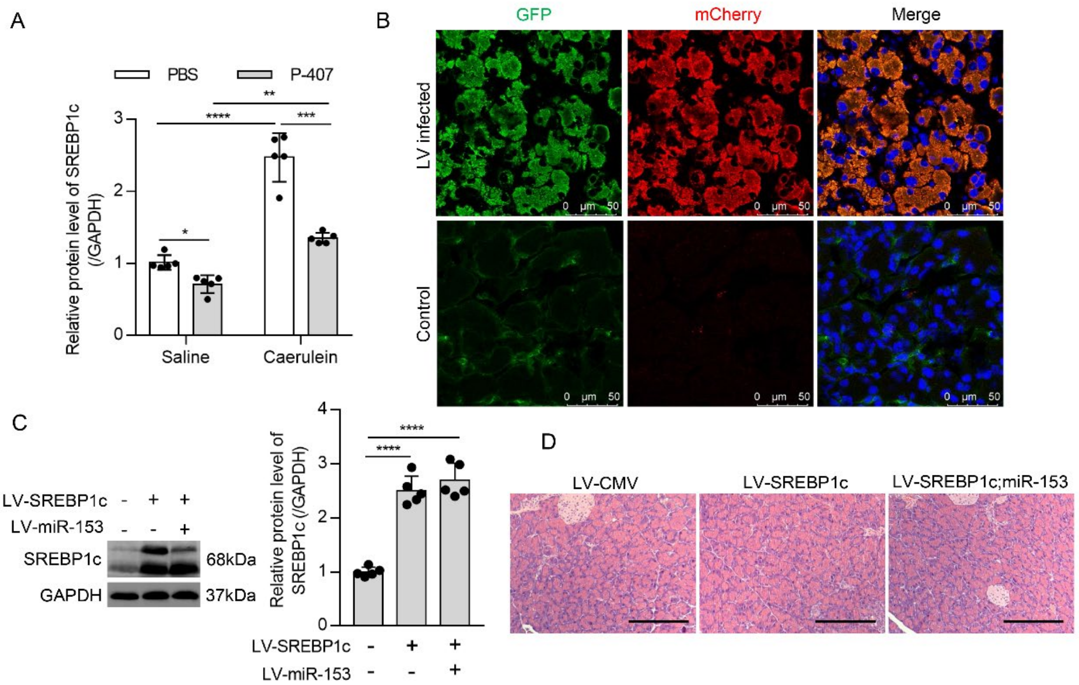

Figure. S7 Lentivirus in situ injection mediates pancreas-specific co-expression of SREBP1c and miR-153 *in vivo.* **A** Statistical quantification of SREBP1c expression in pancreas from figure 4D. **B** Representative image of GFP (green) and mCherry (red) co-staining in the pancreas 30 days after in situ lentivirus injection. Nuclei were labeled with DAPI (blue) (n = 6 mice per group, scale bar = 50 μm). **C** Representative western blots and quantification analysis verifying SREBP1c overexpression in the pancreas of *in situ* lentivirus-injected mice. **D** Representative images of H&E-stained pancreas sections of *in situ* lentivirus-injected mice (n = 6 mice per group, scale bar = 200μm). Data are presented as Mean±SD. (One-way ANOVA).

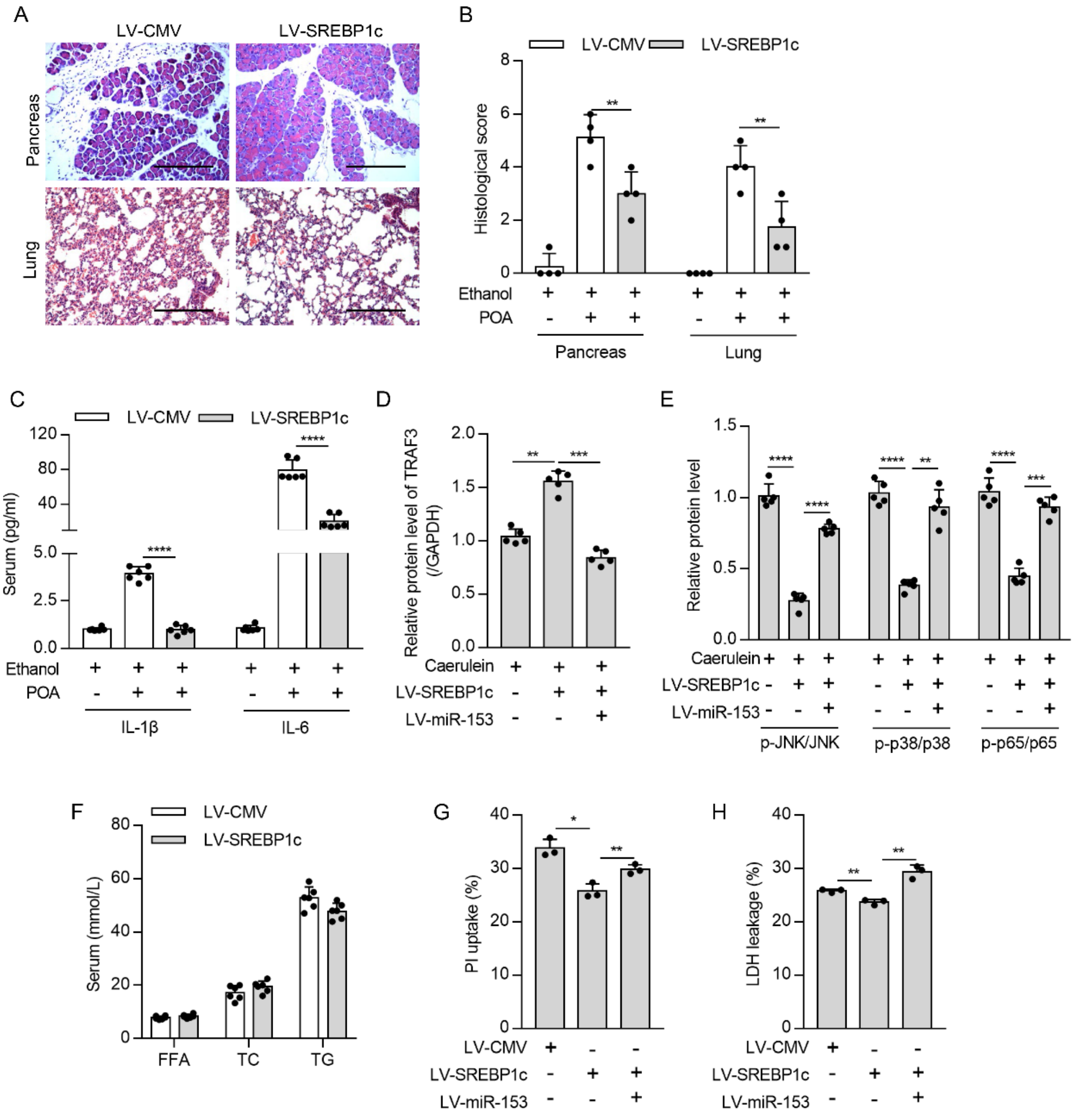

Figure. S8 Overexpression of SREBP1c alleviates histological damages and inflammatory responses in HTG-AP. A Representative images of H&E stained pancreas and lung sections of HTG mice in situ-injected with indicated lentiviruses (*n* = 5-6 mice per group, scale bar = 200μm). **B** Histological score of the pancreas and lung tissues scored by two pathologists independently (10 images per mouse). **C** Serum level of IL-1β and IL-6 of HTG mice in situ-injected with indicated lentiviruses. **D** Statistical quantification of TRAF3 expression in lentivirus-injected pancreas from figure 4J. **E** Statistical analysis showing relative p-JNK, p-p38 and p-p65 expression normalized by total JNK, p38 and p65 respectively in lentivirus-injected pancreas from figure 4J. **F** Serum FFA, TC and TG levels of HTG mice *in situ*-injected with indicated lentiviruses. **G** PI uptake were measured in pancreatic acinar cells isolated from HTG mice in situ-injected with indicated lentiviruses, total cell counts in each well was determined after TritonX-100 treatment. **H** LDH activity in the supernatant of primary acinar cells isolated from HTG mice in situ-treated with indicated lentivirus was measured, an LDH leakage positive control was used to determine percentage of LDH leakage. *P < 0.05, **P < 0.01, ****P < 0.0001. Data are presented as Mean± SD. (One-way ANOVA).

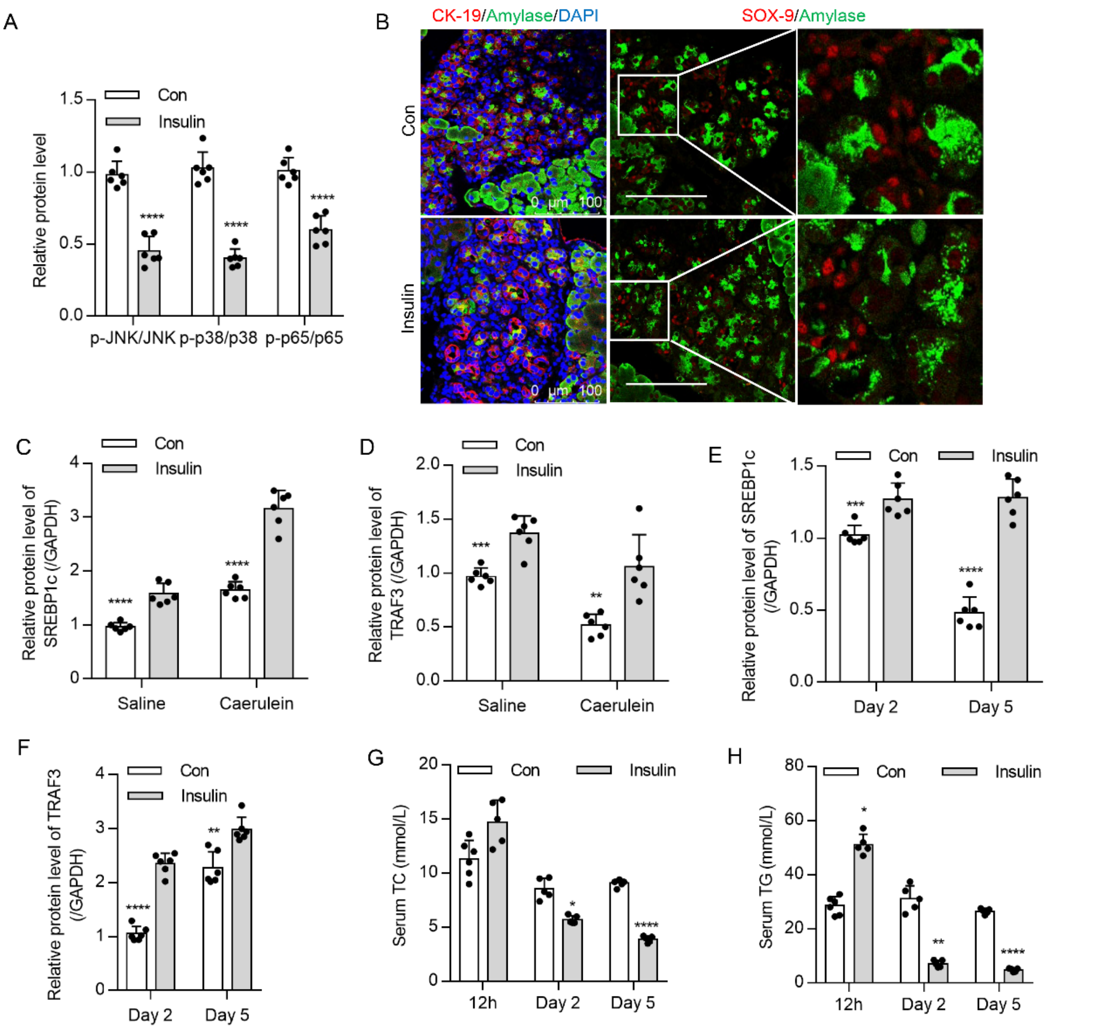

Figure. S9 Effects of insulin in pancreatic repair after HTG-AP. A Statistical analysis showing relative p-JNK, p-p38 and p-p65 expression normalized by total JNK, p38 and p65 respectively in pancreas from figure 6F. **B** Representative images of co-staining of amylase (green) and CK-19 (red) (top), amylase (green) and SOX9 (red) (bottom) at Day 2 after caerulein stimulation in HTG mice with/without insulin treatment. Nuclei were labeled with DAPI (blue). (*n* = 5-6 mice per group, scale bar = 100 μm. **C, D** Statistical quantification of SREBP1c **(C)** and TRAF3 **(D)** expression in AP tissues in figure 6F and control pancreas without caerulein stimulation. **E, F** Statistical quantification of SREBP1c **(E)** and TRAF3 **(F)** expression in pancreas from figure 6K. **G, H** Serum TC (**G**) and TG (**H**) levels in HTG mice with/without insulin treatment. *P < 0.05, **P < 0.01, ****P < 0.0001. Data are presented as Mean±SD. (Student's t-test).

Supplementary Table 1

Gene name		Sequence
Mir-153	RT primer	GTCGTATCCAGTGCAGGGTCCGAGGTATTCGCACTGGATACGACGATCAC
	Forward	CCGGCGGTTGCATAGTCACAAAA
	Reverse	ATCCAGTGCAGGGTCCGAGG
Mir-7	RT primer	GTCGTATCCAGTGCAGGGTCCGAGGTATTCGCACTGGATACGACACAACA
	Forward	GCGCGTGGAAGACTAGTGATTT
	Reverse	AGTGCAGGGTCCGAGGTATT
Mir-129	RT primer	GTCGTATCCAGTGCAGGGTCCGAGGTATTCGCACTGGATACGACATACTT
	Forward	CGAAGCCCTTACCCCAAA
	Reverse	AGTGCAGGGTCCGAGGTATT
Mir-666	RT primer	GTCGTATCCAGTGCAGGGTCCGAGGTATTCGCACTGGATACGACGGCTCT
	Forward	GAGCGGGCACAGCTGTG
	Reverse	AGTGCAGGGTCCGAGGTATT
Mir-802	RT primer	GTCGTATCCAGTGCAGGGTCCGAGGTATTCGCACTGGATACGACCTGAGT
	Forward	CGCGACGGAGAGTCTTTGTC
	Reverse	AGTGCAGGGTCCGAGGTATT
Rnu6(mouse)	RT primer	CTCAACTGGTGTCGTGGAGTCGGCAATTCAGTTGAGAAAAATAT
	Forward	CAAGGATGACACGCAAA
	Reverse	TCAACTGGTGTCGTGG
Rnu6(rat)	Forward	CTCGCTTCGGCAGCACA
	Reverse	AACGCTTCACGAATTTGCGT
Srebf1	Forward	CTGACAGGTGAAATCGGCG
	Reverse	GCATGTCTTCAAATGTGCAATCC
Traf3	Forward	CAGCCTAACCCACCCCTAAAG
	Reverse	TCTTCCACCGTCTTCACAAAC
Il1b	Forward	TTGACGGACCCCAAAAGAT
	Reverse	GAAGCTGGATGCTCTCATCTG
Il6	Forward	TTCATTCTCTTTGCTCTTGAATTAGA
	Reverse	GTCTGACCTTTAGCTTCAAATCCT
Tnf	Forward	TCTCTTCAAGGGACAAGGCTG
	Reverse	ATAGCAAATCGGCTGACGGT
Gapdh	Forward	AGGTCGGTGTGAACGGATTTG
	Reverse	TGTAGACCATGTAGTTGAGGTCA
